# Comparing large-scale computational approaches to epidemic modeling: Agent-based versus structured metapopulation models

**DOI:** 10.1186/1471-2334-10-190

**Published:** 2010-06-29

**Authors:** Marco Ajelli, Bruno Gonçalves, Duygu Balcan, Vittoria Colizza, Hao Hu, José J Ramasco, Stefano Merler, Alessandro Vespignani

**Affiliations:** 1 Predictive Models for Biomedicine and Environment, Bruno Kessler Foundation, Trento, Italy; 2 Center for Complex Networks and Systems Research, School of Informatics and Computing, Indiana University, Bloomington, IN 47408, USA; 3 Computational Epidemiology Laboratory, Institute for Scientific Interchange Foundation, Turin, Italy; 4 Department of Physics, Indiana University, Bloomington, IN 47408, USA; 5 Pervasive Technology Institute, Indiana University, Bloomington, IN 47404, USA

## Abstract

**Background:**

In recent years large-scale computational models for the realistic simulation of epidemic outbreaks have been used with increased frequency. Methodologies adapt to the scale of interest and range from very detailed agent-based models to spatially-structured metapopulation models. One major issue thus concerns to what extent the geotemporal spreading pattern found by different modeling approaches may differ and depend on the different approximations and assumptions used.

**Methods:**

We provide for the first time a side-by-side comparison of the results obtained with a stochastic agent-based model and a structured metapopulation stochastic model for the progression of a baseline pandemic event in Italy, a large and geographically heterogeneous European country. The agent-based model is based on the explicit representation of the Italian population through highly detailed data on the socio-demographic structure. The metapopulation simulations use the GLobal Epidemic and Mobility (GLEaM) model, based on high-resolution census data worldwide, and integrating airline travel flow data with short-range human mobility patterns at the global scale. The model also considers age structure data for Italy. GLEaM and the agent-based models are synchronized in their initial conditions by using the same disease parameterization, and by defining the same importation of infected cases from international travels.

**Results:**

The results obtained show that both models provide epidemic patterns that are in very good agreement at the granularity levels accessible by both approaches, with differences in peak timing on the order of a few days. The relative difference of the epidemic size depends on the basic reproductive ratio, *R*_0_, and on the fact that the metapopulation model consistently yields a larger incidence than the agent-based model, as expected due to the differences in the structure in the intra-population contact pattern of the approaches. The age breakdown analysis shows that similar attack rates are obtained for the younger age classes.

**Conclusions:**

The good agreement between the two modeling approaches is very important for defining the tradeoff between data availability and the information provided by the models. The results we present define the possibility of hybrid models combining the agent-based and the metapopulation approaches according to the available data and computational resources.

## Background

Computational approaches for the detailed modeling of epidemic spread in spatially-structured environments make use of a wide array of simulation schemes [1,2]. In recent years, two major classes of methodologies emerged in the simulation of influenza-like illnesses (ILIs) and other emerging infectious diseases. The first one is the very accurate epidemic description with agent-based models, which keep track of each individual in the population in an extremely detailed way [3-14]. The second scheme relies on metapopulation structured models that consider in a detailed way the long range mobility scheme at the inter-population level while using coarse-grained techniques at the intra-population level [15-25]. Agent-based models provide a very rich data scenario, but the computational cost and, most importantly, the need for very detailed input data has limited its use to country level [6-11] or continental level [12] scenarios so far. On the opposite side, the structured metapopulation models are fairly scalable and can be conveniently used to provide worldwide scenarios and patterns with thousands of stochastic realizations [18,20,21,23-25]. While on the one hand, the level of information that can be extracted in this latter case is less detailed than those of agent-based models, the spatial and temporal ranges and the number of realizations that can be computationally analyzed is much larger. Also, the amount of data to be integrated is less massive than in agent-based frameworks. From this perspective, it is clearly important to assess the level of agreement that the two different approaches can provide with respect to the quantities accessible, the respective data needed, and the computational costs associated with both approaches.

Comparing different models is often a hard task. While on one side one would like to assess the role of the differences inherent to each of the modeling frameworks, it is important to establish a common ground between the two frameworks in order to discount unwanted effects due to different parameterization (see for example the discussion of the estimation of the reproductive number for the SARS epidemic obtained from a variety of models in Ref. [26]). An attempt in this direction was presented in Ref. [10] where three individual-based models with different assumptions and data - one at the description level of a city and two at the description level of a country - were compared through their predictions in the case of interventions against a new pandemic influenza strain. However, the comparison was constrained to each model's assumptions and to the available simulated scenarios, without explicitly defining a common set of parameters and approximations to be shared by all models. The low transmission scenario was compared in different models by using different values for the reproductive number, with the risk of not being able to discount the effect of this difference in the obtained results.

Here we provide for the first time a side-by-side comparison of the results obtained at the level of a single country by using state-of-the-art structured metapopulation and agent-based models developed independently and employed in previous works to analyze pandemic events [8,9,11,12,18,24,25,27]. Both models have been used in realistic scenarios [14,27] and incorporating actual data in relation to the H1N1 pandemic [24,28]. However, comparing simulation results with real data would require a thorough discussion and analysis of the disease parameters, the identification of the initial conditions, the assessment of the reliability of reporting and notification systems that are the sources of the empirical data. This is not the object of this paper. Instead, we focus on the differences generated by the two modeling approaches.

For the sake of clarity we compare the two models in a clean synthetic experiment of a hypothetical pandemic event for which we assume the same parameterization with regards to the modeling aspects that the models share, such as disease progression and initial conditions. The country used for the study is Italy, a large European country that provides the necessary geographic and population heterogeneity to assess the models' performance in the case of highly-structured populations. The two approaches access different granularity levels and we use as a comparison the finer spatial resolution accessible by both models. This allows us to analyze 39 major subpopulations and project data at the administrative level of municipality.

We find that both models, despite the difference in the data integration and model structure, provide epidemic profiles with spatio-temporal patterns in very good agreement. The epidemic size profile shows an expected overall mismatch of 5-10% depending on the reproductive rate, which is induced by the homogeneous assumption of the metapopulation strategy. Breaking down data at the level of age-structured compartments shows that both models provide very similar results with the exception of the elderly population (60 + age bracket), which show larger epidemic sizes in the metapopulation approach. The good agreement of the two approaches reinforces the message that computational approaches are stable with respect to different data integration strategies and modeling assumption. On the other hand, the agent-based model approach may access information not available to the coarser metapopulation approach, and relevant for individually based or targeted intervention measures. This is at the price of a higher computational cost and the availability of fine resolution data, whereas the metapopulation approach is less dependent on detailed data and is computationally cheaper. The presented results hint to the possibility of combining the two methodologies in order to devise multiscale approaches that use the data parsimony of the metapopulation approaches at the global level and the high resolution of the agent-based model in specific locations of interest where detailed data are available.

## Methods

### The agent-based modeling scheme

The considered agent-based model is a stochastic, spatially-explicit, discrete-time, simulation model where the agents represent human individuals. The infection can spread among individuals through contacts with household members, school and workplace colleagues, and by random contacts with the general population [5,6]. One of the key features of the model is the characterization of the network of contacts among individuals based on a realistic model of the socio-demographic structure of the Italian population [8,9].

**Figure 1:**
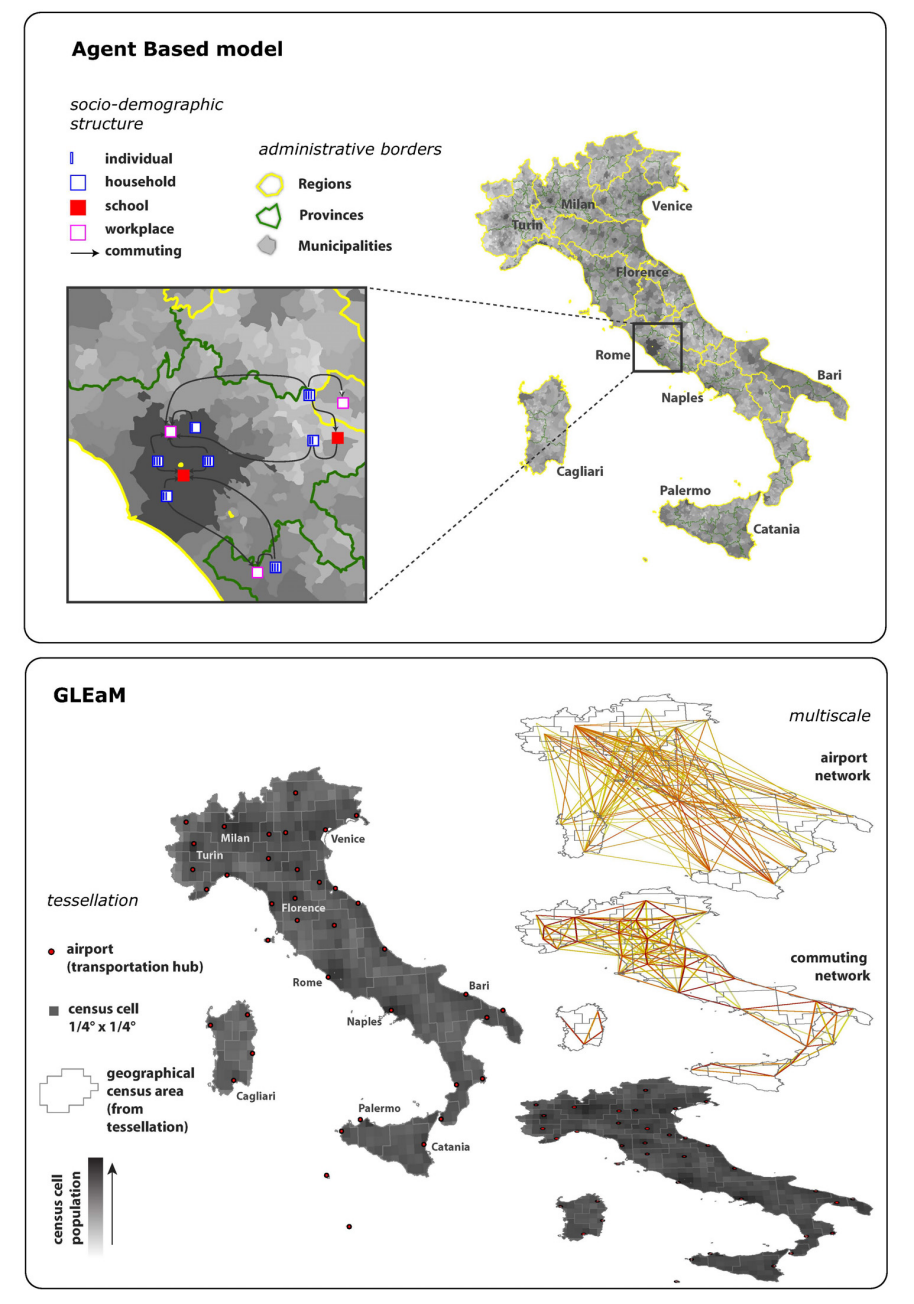
**Agent-based model and GLEaM**. Top: The agent-based model is a stochastic and spatially-explicit simulation model where the agents represent individuals. The basic spatial structures considered in the model are the municipalities. The force of infection in the general population is assumed to decrease with the geographic distance among municipalities. The dependence on the distance is modeled by a gravity model as derived by the analysis of data on travel to school or work (grouped by all hierarchical administrative levels, from the national level down to the municipality level). The inset shows the explicit representation of individuals in the model enabling the simulations of the most important contacts for diseases transmission, i.e. household, school, and workplace contacts. The spatial spread of the epidemic is determined by i) transmission in the general population at the national scale and ii) transmission in schools and workplaces at a more local scale. Bottom: GLEaM, GLobal Epidemic and Mobility model. The world surface is represented in a grid-like partition where each cell — corresponding to a population value — is assigned to the closest airport. Geographic census areas emerge that constitute the subpopulations of the metapopulation model. The demographic layer is coupled with two mobility layers, the short-range commuting layer and the long-range air travel layer.

Population data for Italy — 56,995,744 individuals — is obtained from the census of 2001 [29] (382,534 census sections). According to the administrative borders of the country under study, the population is hierarchically grouped by municipalities (8,101), provinces (103) and regions (20), which also provide the spatial structure of the model (see Figure 1 and the Additional 1 File for details). Census data on age structure and frequencies of household type and size are jointly used with specific survey data on Italian households [30] to assign age and to co-locate individuals in households. For each municipality, an appropriate number of households (and individuals) is generated to match the actual resident population.

Demographic, school, and industry census data from 2001 [31,32] are used for assigning an employment category (student, worker, or unemployed/retired) to individuals on an age basis. The legal working age in Italy is 15. Data on school attendance is available for individuals aged ≤ 14 years for any one-year age class. For individuals aged ≥ 15 years, data on school attendance and employment rate is available for any one-year age class. An employment category is assigned to any individual by sampling from the age-dependent distribution of the frequencies of employment as obtained from the analysis of the data described above. In the model we first assign a size to schools and workplaces on the territory (schools and workplaces are spatially-distributed proportionally to the population). Then we locate students and workers in the different places in such a way that the probability density function of travel distances complies with available commuting data for Italy.

Data on the proportion of individuals with age ≥ 15 working or attending school in the same municipality of residence is available for each municipality, together with the number of individuals traveling either to a municipality of the same province they live in, outside the province but within the same region, and outside the region. For determining the probability of commuting from municipality to municipality we use a general gravity model used in transportation theory [33,34] of the form(1)

where *N*_*i *_and *N*_*j *_are the number of individuals living in municipality *i *and *j *respectively, *d*_*ij *_is the distance between the two municipalities, *θ *is a proportionality constant, *τ*_*f *_= 0.28 and *τ*_*t *_= 0.66 tune the dependence of dispersal on donor and recipient sizes, and *ρ *= 2.95 tunes the dependence on the distance. Here we assume a power law functional form for the distance dependence, as in [35], although other functional forms — such as an exponential decay — can be considered [25,33,34].

The epidemic transmission model assumes that the infection can be transmitted within households, schools, workplaces, and by random contacts in the general population. Any susceptible individual *i *at any time *t *of the simulation has a probability(2)

of being infected, where Δ*t *is the time step of the simulation and *λ*_*i *_is the instantaneous risk of infection. The latter is the sum of the risks coming from the three sources of infection: (1) contacts with infectious members of the household, (2) contacts with infectious individuals working in the same workplace or attending the same school, and (3) random contacts with infectious individuals in the population. While we assume homogeneous mixing in households, schools and workplaces, random contacts in the general population are assumed to depend explicitly on distance. Specifically, the contribution to the force of infection determined by an infectious individual *k *is weighted by the following kernel(3)

a decreasing function of the geographical distance *d*_*ik*_. Parameters *a *and *b *were optimized by employing Eq. (3) for generating a synthetic population of commuters such that the resulting probability density function of travel distances matches that obtained by using the gravity model of Eq. (1). The estimated parameters are *a *= 3.8 *km *and *b *= 2.32. As in [5,8,9], the model is parameterized so that 33% of transmission occurs in households, 33% in schools and workplaces and 33% in the general community. The epidemic transmission dynamics is based on an ILI compartmentalization as described in the subsection Models calibration (full details on the detailed formulation of the model are provided in the Additional File 1).

### Metapopulation modeling scheme

The Global Epidemic and Mobility (GLEaM) model is based on a metapopulation approach [15-21] in which the world is divided into geographical regions defining a subpopulation network where connections among subpopulations represent the individual fluxes due to the transportation and mobility infrastructure [24,25]. Infection spread occurs inside each urban area and is described by compartmental schemes in which the discrete stochastic dynamics of the individuals among different compartments depends on the specific etiology of the disease and the containment interventions considered. GLEaM integrates a highly detailed population database worldwide with the air transportation infrastructure and short-range mobility patterns [24,25]. Air travel mobility is obtained from the International Air Transport Association (IATA [36]) database that contains the list of worldwide airport pairs connected by direct flights and the number of available seats on any given connection [37]. The resulting worldwide air-transportation network is a weighted graph composed of 3,362 vertices denoting airports in 220 different countries and 16,846 weighted edges whose weight, *ω*_*jl*_, represents the number of passengers flying between airports *j *and *l*, accounting for 99% of worldwide traffic. Each airport is associated to a geo-referenced census area as obtained from a Voronoi tessellation on the population database [25]. GLEaM is based on the high-resolution population database of the "Gridded Population of the World" project of SEDAC [38] (Columbia University), which estimates the population with a granularity given by a lattice of cells covering the whole planet at a resolution of 15 × 15 minutes of arc. We define the geographical census areas centered on IATA airports by assigning each cell to the closest airport as long as the distance between the center of the cell and the airport is less than 200 km. This is the characteristic length scale of the cell/airport distribution as well as the scale for the intensity of the ground commuting flows [24]. Such a procedure divides Italy into *39 *distinct areas (subpopulations) that define the metapopulation structure we use. A schematic illustration of the model and of the layers considered is reported in Figure 1.

The geo-referenced nature of the subpopulations allows for the integration of short-scale mobility between adjacent subpopulations into the model. GLEaM considers commuting and mobility patterns of various means of land transportation (bus, cars, train, etc.). National commuting data available at administrative levels are then mapped into the geographic census areas obtained from the tessellation procedure [25,33,34]. In the present study we use real mobility data for Italian municipalities as provided by the Italian National Statistics and Census Bureau (ISTAT) to obtain the commuting flows among the census areas defining the Italian subpopulations.

GLEaM is fully stochastic and can simulate the long-range mobility of individuals from one subpopulation to another subpopulation by means of the airline transportation network in a manner similar to the models presented in Refs. [15-25]. In particular, in each city *j *the number of passengers traveling on each connection *j *→ *l *at time *t *defines a set of stochastic variables that follow a multinomial distribution [22]. The calculation can be extended to include transit traffic as well, e.g. up to one connection flight [39]. Short-range, multi-modal transportation between subpopulations is modeled with a time-scale separation approach that defines an effective force of infection in connected subpopulations based on the real commuting flow data between adjacent subpopulations integrated in the model [25,40,41]. The discrete nature of individuals is also preserved in compartmental transitions and in short-range mobility processes. The transmission model within each geographical census area follows an ILI compartmentalization common to the agent-based model, as shown in the following section. The contagion process (i.e. the generation of new latent individuals from the contact of infectious and susceptible individuals) and the spontaneous transitions (e.g. from latent to infectious or from infectious to recovered) are modeled with multinomial distributions. The actual expressions used for the force of infection contain several terms, as they have to discount non-traveling infectious individuals and second order terms generated by the interactions of individuals from neighboring subpopulations. Here we also introduce the age structure of the population by defining a contact matrix specifying the force of infection across different age brackets. We adopt the contact matrix formalism and the age classes defined by Wallinga and collaborators [42]. In this case the basic reproduction number *R*_0 _is determined by the largest eigenvalue of the modified next generation matrix. The full derivation of the epidemic model and its implementation is reported in the Additional File 1.

### Models calibration

**Figure 2:**
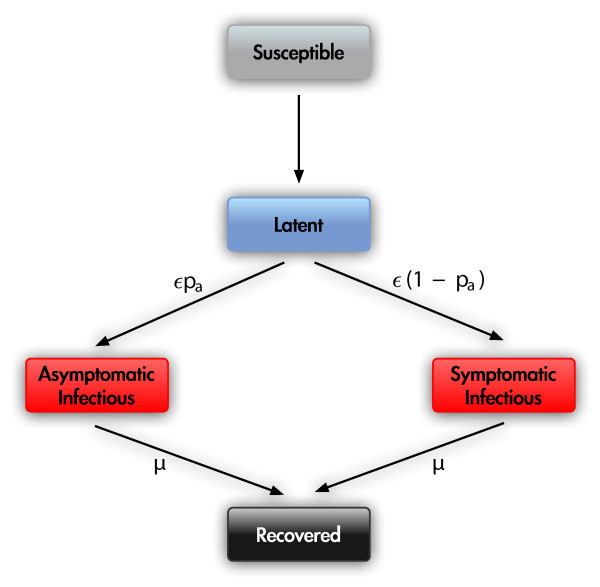
**Disease compartmental structure**. Diagram flow of the infection transmission structure adopted by both models. The transition from the susceptible class to the latent class is induced by the interaction between the susceptible individuals and the infectious individuals (see text).

In order to study the effect of the assumptions related to the different approaches exclusively, we align the set of parameters for the disease transmission model and the initial conditions in both models (see Table 1). The agent-based and metapopulation models are stochastic, spatially structured, and based on discrete time simulations. Though the social and mobility structure changes across the models, both GLEaM and the agent-based model are based on the same transmission dynamics. The models adopt a compartmentalization for an ILI defined in terms of susceptible (*S*), latent (*L*), asymptomatic infectious (*I*^*a*^), symptomatic infectious (*I*), and permanently recovered/removed (*R*) (see Figure 2).

**Table 1:** Model parameters

Model parameters	GLEaM	Agent-based
***Initial conditions***		
Origin of pandemic	Hanoi	Hanoi
Arrival of infection in country	Simulating global pandemic	Provided by GLEaM

***Transmission dynamics (common to both models)***		
Basic reproductive ratio, *R*_*0*_		1.9 [1.5, 2.3]
Average latency period, ε^-1^		2.0 days
Average infectious period, μ^-1^		3.0 days
Probability of asymptomatic disease, *p*_*a*_		33%
Reduction in disease transmission due to asymptomatic disease, *r*_*β*_		50%

***Mobility***		
Long/short range travel	Explicit air travel with 70% daily average occupancy of flights.	Commuting model used for geographically locating schools and workplaces.
	Implicit commuting through effective force of infection with τ = 3 day^-1 ^return rate and real commuting fluxes.	Implicit within country through random contacts in the general population.
Impact of symptomatic disease on individual behaviour	Stop travelling when ill with probability 1-*p*_*t *_= 50%	Reduction in school and work attendance, ranging from 90% in daycare centers to 50% in workplaces.

Model assumptions and parameter values used as baseline [and sensitivity analysis] are summarized both for GLEaM and the agent-based model.

A susceptible individual in contact with a symptomatic or asymptomatic infectious person can contract the infection and enter the latent compartment where he is infected but not yet infectious. The transmission occurs at different rates that take into account the reduced infectiousness of asymptomatic individuals and additional effects, e.g. those induced by absenteeism that are considered in the agent-based model (a full discussion is reported in the Additional File 1). At the end of the latency period, each latent individual becomes symptomatic with probability 1 - *p*_*a *_or becomes asymptomatic with probability *p*_*a*_. All infectious individuals recover permanently (i.e. become immunized from further infection) and enter the recovered compartment at rate *μ*. We fix the average latency period *ε*^-1 ^= 2 days and the average infectious period *μ*^-1 ^= 3 days [4,18,43] equal in the two models. Given that infection has occurred, both GLEaM and the agent-based model assume that individuals become asymptomatic with probability *p*_*a *_= 0.33 [4,18,43], with a relative infectiousness equal to *r*_*β *_= 0.5. In addition, both models assume that clinical disease affects individual behavior. GLEaM assumes that symptomatic individuals avoid traveling with probability 1- *p*_*t *_= 0.5 [18,43], whereas the agent-based considers the reduction of school and work attendance [5,6,8] (see the Additional File 1 for details). The spreading rate of the disease is governed by the basic reproduction number (*R*_0_) which is defined as the average number of infected cases generated by the introduction of a typical infectious person into a fully susceptible population [44]. For the proposed compartmentalization, its value can be obtained for GLEaM by evaluating the largest eigenvalue of the Jacobian or next generation matrix of the infection dynamics in a disease-free equilibrium [45], yielding *R*_0 _= *βμ*^-1^(1 - *p*_*a *_+ *r*_*β*_*p*_*a*_) if the age structure is not considered. In the case of the agent-based model, it is computed as(4)

where *r *is the intrinsic growth rate of the simulated epidemic.

The two models are calibrated to the same value of the reproductive number *R*_0_. In addition, GLEaM and the agent-based model are also dynamically calibrated in that they share exactly the same initial/boundary conditions. GLEaM is defined at the worldwide scale and allows the study of an emerging epidemic under a variety of geographical and temporal initial conditions based on any geographical census area of the model at any time of the year. The agent-based model is defined at the level of the country, and, as in other individual-based stochastic simulations describing the scale of a given region [3,6,7], it is based on the importation of cases from abroad. The case importation is generally modeled through a global unstructured SEIR compartmental model that simulates the epidemic worldwide and feeds the country of interest through cases arriving at the international airports proportional to the traffic of the airports.

Several procedures can be modeled, including both those with stationary initial conditions in which the simulations let the epidemic progress after the first seeding has occurred with no additional importation of cases [9], and those with dynamic initial conditions in which the importation of cases is not stopped by the beginning of the epidemic in the country under study [6,8]. In order to align GLEaM and the agent-based model under the same initial conditions, we assume dynamic importation of cases in the agent-based model as provided by GLEaM. We choose Hanoi, Vietnam, as the seed of the epidemic for GLEaM and study the geotemporal spreading pattern of the epidemic at the worldwide scale. The number of infected individuals imported into Italy at each international airport is tracked in time in each stochastic realization and provides the set of the dynamic initial conditions for the agent-based model. This approach allows us to study the evolution of the epidemic in Italy with the two models side-by-side, discounting the effects that relate to different seeding at the boundary of the country.

Here we study a pandemic baseline scenario, assuming no seasonality as in Refs. [6-8], taking on three values for the reproductive number, *R*_0 _= 1.5, 1.9, and 2.3, in the range of expected values for a newly-emerging influenza pandemic as based on estimates for previous pandemics [15,46]. We do not implement intervention strategies because our aim is to explore the effect of two different modeling frameworks in shaping the epidemics, assessing analogies and differences induced by each model's assumptions.

All results in the following section are based on 50 stochastic realizations per model, each realization feeding the two models with equal dynamic initial conditions. Results are reported at different resolution scales, including the country level, the geographical census areas around major transportation hubs, and the smallest scale of municipalities. Italy includes *8,101 *municipalities that are grouped in *39 *GLEaM geographical census areas.

## Results

### Country scale

**Figure 3:**
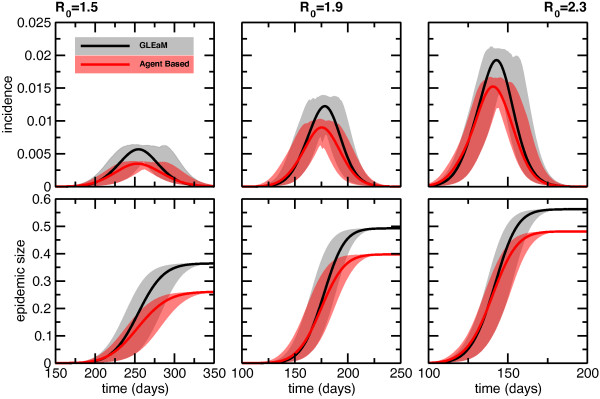
**Comparison of the epidemic incidence and size**. Incidence profiles and epidemic size for GLEaM and the agent-based model at the global level. Time is expressed in days since the first importation of infected individuals in Italy. Results for three values of the reproductive number are shown from left to right: *R*_0 _= 1.5, *R*_0 _= 1.9, *R*_0 _= 2.3. Average profiles (lines) and 95% CI (shaded areas) are shown.

Figure 3 shows the timeline of the incidence profile and of the epidemic size obtained with GLEaM and with the agent-based model. Time is expressed in days, and the first importation of infectious individuals into Italy is used to synchronize the two models. Thanks to the initial alignment, Figure 3 shows the epidemic unfolding side-by-side in the same time window explored by the two models, so that it is possible to assess the timing and synchronization of the simulated epidemics. The incidence profiles show that on average the two temporal patterns are in very good agreement, despite the very different data integration and assumptions of the two models. The two peaks are just a few days apart from each other, with GLEaM on average reaching the peak of the epidemic slightly later than the agent-based model. The value of the epidemic incidence at the peak in the simulations obtained with the agent-based model is lower than in the simulations with the GLEaM model. This difference has to be expected since we are comparing an individual-based approach with a spatially-structured model based on an assumption of homogeneous transmission rates for the interactions of people in the subpopulations. Indeed, as observed in earlier works, models with heterogeneous transmission rates across population groups present different attack rates - usually lower - than those with homogenous mixing, even for the same overall value of *R*_*0 *_(See for instance the discussion in [47,48] and references therein). Changes in attack rates and even epidemic thresholds are also observed when the full interaction pattern of individuals is considered [49-51]. While the GLEaM model just considers the spatial structure and the age structure, the agent-based model used here is highly structured and considers households, schools, etc. The two models therefore are expected to present different attack rates. The difference in the peak amplitudes decreases for increasing values of the reproductive number and the same effect is also evident from the curves of the epidemic size. At the end of the epidemic outbreak, the average size predicted by GLEaM ranges from 36% for *R*_*0 *_= 1.5 to 56% for *R*_*0 *_= 2.3, as compared to the one observed in the agent-based model which ranges from 26% for *R*_*0 *_= 1.5 to 49% for *R*_*0 *_= 2.3, with an absolute difference of about 10% for *R*_*0 *_= 1.5 and 7% for *R*_*0 *_= 2.3. Fluctuations are comparable in the two models, as shown by the shaded areas around the average values, representing the 95% reference ranges obtained from the stochastic runs.

The subpopulation structure of GLEaM and its coupling with mobility processes preserves accurate timing in different geographical areas. However, when attack rate is considered we still see differences, as the household and workplace structure are important in differentiating the impact on different age brackets. GLEaM includes a spatial substructure that subdivides the global populations into subpopulations around major transportation hubs. Inside each census area the subpopulation is divided into age classes. The frequency of interaction among individuals in different age classes is governed by a specific matrix such that within each age class the individuals are all considered equivalent and a homogenous assumption is used for the evaluation of the force of infection. The agent-based model is more refined in the definition of the social/spatial/age structure in the population, being defined at the level of the single individual. In this case each individual is tagged with the appropriate social bracket by assigning the household structure, workplace size, etc.

As we will see in the next sections, the main differences in the two models are observed for the 60+ age class. Indeed, this is the age class with the most marked differences in household structure and workplace habits; such differences cannot be taken into consideration in the metapopulation level. It is however difficult to state which of the two predictions is the most accurate. On one hand the high level of realism of the agent-based model should make the prediction reliable. On the other hand this high realism is not free of modeling assumptions, as for instance in the definitions of Eqs. (1) and (3). The correct value should be in between the prediction of the models, as supported by the fact that the difference between the models decreases as *R*_*0 *_increases, with the models converging to the same value for the attack rate. For large *R*_*0 *_in fact, the local epidemics - in census areas for GLEaM, and in households/workplaces in the agent-based model - become more widespread across all the layers of the population and thus the differences in the population structure are less relevant. In the Additional File 1 we also report the results for a simple single SLIR population model aligned with the agent-based and metapopulation models. As expected such a simple model is not able to recover the variability of the incidence profile and the final attack rate of the epidemic.

**Figure 4:**
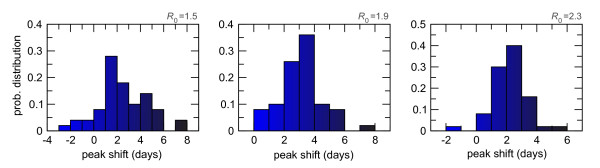
**Activity peaks difference in the two models**. Histogram of the activity peak difference *(T*_*GLEaM *_- *T*_*AB*_) (measured in days) between GLEaM and the agent-based model at the global level. The histogram is obtained by comparing each pair of stochastic realizations in the two models and considering negative and positive differences when the GLEaM activity peak occurs before or after the agent-based model, respectively. Results for three values of the reproductive number are shown from left to right: *R*_0 _= 1.5, *R*_0 _= 1.9, *R*_0 _= 2.3.

The peak delay between the two models is defined as the absolute difference between the activity peak time *T*_*GLEaM *_and *T*_*AB *_of the metapopulation and agent-based models, respectively. The difference *(T*_*GLEaM *_- *T *_*AB*_) is expressed in days and calculated for each pair of stochastic realizations. Figure 4 shows the probability distributions of this quantity, calculated for the three values of *R*_0 _explored. We consider both negative and positive differences corresponding to one model anticipating the other or vice versa. GLEaM more likely reaches the peak later than the agent-based model, with a most probable delay of about 2-4 days, explaining the very good agreement in the timing observed in Figure 3. Fluctuations around these values are reduced for increasing values of *R*_0_, being -3 to 8 days for *R*_0 _= 1.5 and -2 to 6 days for *R*_0 _= 2.3, showing how higher transmission scenarios would lead to more synchronized epidemics in the two models.

### Census area scale

**Figure 5:**
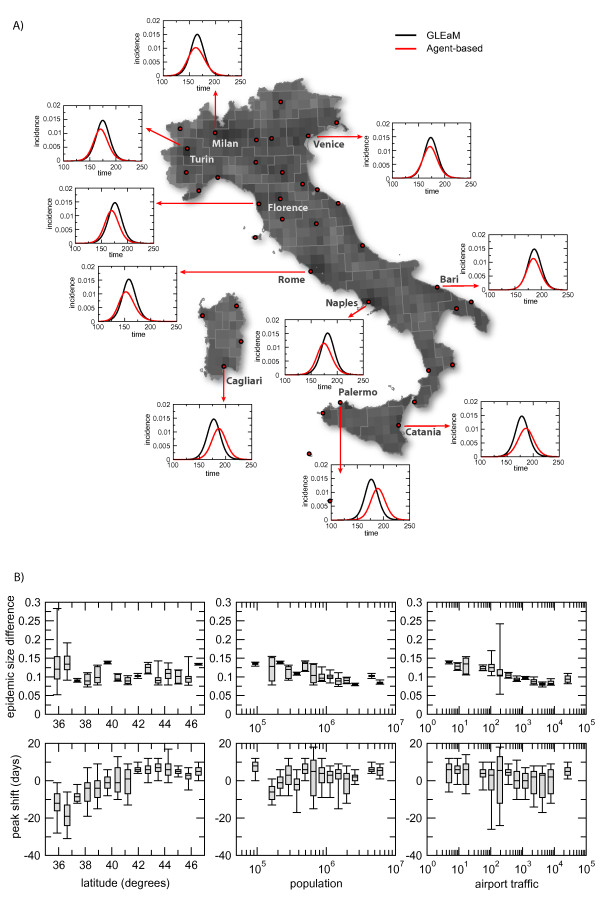
**Epidemic profiles and geography**. Geographic variation of the epidemic profiles for GLEaM and the agent-based model at the level of the major urban areas in Italy: a) profiles for a selected number of Italian subpopulations distributed from North to South and in the Islands. Time is expressed in days since the first importation of infected individuals in Italy. Average profiles for the scenario with *R*_0 _= 1.9 are shown; b) difference of the epidemic size as a fraction the population size (top row) and peak shift measured in days (bottom row) between GLEaM and the agent-based model at the level of GLEaM geographical census areas as functions of: the latitude of the geographical census area (left); its population size (center); and the traffic of the airport associated to the geographical census area (right). Results for *R*_0 _= 1.9 are shown.

Given the high spatial definition of both models, it is possible to further investigate differences in the observed epidemic patterns by looking at the results obtained in different spatial regions of Italy. In particular, we focus on the geographical census areas defined in GLEaM and aggregate the simulation results of the agent-based model from the scale of municipalities to the scale of the geographical census areas. Figure 5A reports the average incidence profiles of a selected number of geographical census areas in Italy distributed from North to South, and the large islands. Results are shown for *R*_0 _= 1.9, whereas additional results for the other two values explored are reported in the Additional File 1. The plots show heterogeneous variations in the comparison of the profiles, with geographic census areas where the two models are synchronized and others in which the agent-based profile is shifted before or after the GLEaM model by a few days. Also, the differences in the peak amplitude vary across the country. We thus explored possible relations between the observed differences in the timing and size of the epidemic and some features at this resolution scale that are common to both models. In particular, we considered: (i) the North-South position of the geographic census area as indicated by the latitude of its centroid, around which the area was defined in GLEaM through the tessellation procedure; (ii) the population size of the geographic census area; and (iii) the airline traffic of the geographic census area, defined as the number of passengers per day traveling through its airports.

Results in Figure 5B show that the differences in the epidemic size tend to be stable from North to South, and to decrease with increasing population size and increased airport traffic. This can be explained by the fact that larger numbers in population and traffic (on average large population sizes are associated to large traffic airports [22]) smooth out differences and the effect of fluctuations, which are instead more pronounced in populations of small size. If we look at the timing, we observe that there is a pronounced anticipation of the GLEaM model with respect to the agent-based model in the Southern regions (especially in the Islands), reaching a good synchronization in the Center and a stationary small delay in the North of the country. Because of the stationary behaviors in the relations between the peak shift and the population size or airport traffic of the geographical census areas, the results observed with respect to latitude appear to indicate a genuine difference between the two frameworks. Both models consider commuting patterns - GLEaM integrates the commuting network among geographic census areas obtained from the Italian origin-destination commuting data, and the agent-based model integrates a synthetic commuting network among municipalities reproducing the statistics of commuters throughout the country from coarse-grained information on destination data. Though built on different levels of detail, both commuting networks are expected to reproduce the geographical fluctuations observed in the mobility of the Italian population, with a percentage of commuters increasing from 15% in Southern Italy to 60% in Northern Italy. Long distance travel seems instead to be responsible for the observed behavior in the peak shift vs. the latitude. The distance kernel for random contacts in the population considered in the agent-based model might be unable to reproduce some of the complex properties that are found in the air travel flows with North-South heterogeneities. In this respect, the introduction of long-distance travel in the agent-based model [9] could contribute to smooth out differences.

### Municipality scale

**Figure 6:**
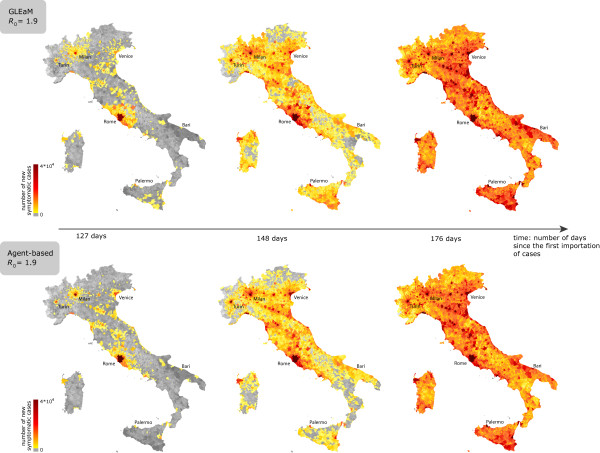
**Geotemporal spreading pattern of the epidemic**. Comparison of the spatial epidemic evolution in GLEaM (top) and in the agent-based model (bottom) at three different snapshots of the simulation for *R*_0 _= 1.9. From left to right snapshots show: 127 days, 148 days, and 176 days after the first importation of infected individuals in Italy. Maps reproduce the average number of cases at the resolution scale of the Italian municipalities.

By increasing the spatial resolution even further, it is possible to monitor the geotemporal spread of the disease at the level of the 8,101 municipalities in the country. The results by GLEaM at the level of the geographic census areas are mapped into the administrative boundaries of the municipalities to be comparable with the simulation results produced by the agent-based model. The observed epidemic pattern is shown in Figure 6 for three different snapshots of the simulations in terms of average values of the new number of clinical cases per municipality. The visualization confirms the above results, showing a very good agreement of the geographic distribution of cases at the finest resolution scale available.

### Age class breakdown

**Figure 7:**
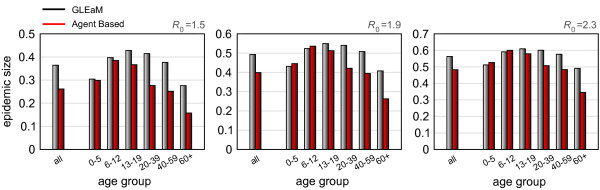
**Cumulative cases in different age brackets**. Comparison of epidemic size by age group between GLEaM and the agent-based model for three values of the reproductive number: *R*_0 _= 1.5 (left), *R*_0 _= 1.9 (center), and *R*_0 _= 2.3 (right).

The age structure of GLEaM comprises 6 classes of age, namely 0-5, 6-12, 13-19, 20-39, 40-59, and 60 + years old. Results on the incidence by age as obtained by the agent-based model have been aggregated according to the age structure of GLEaM, which allows us to compare the simulations' results broken down by age classes. Figure 7 shows the epidemic size by age class as obtained by the two models for the three values of *R*_0 _investigated. In all cases the agreement is higher in the younger age classes (0-5, 6-12, and 13-19 years old), and deviations start to be more pronounced for the young adult, adult, and older age classes. However, as seen before when considering all age classes, deviations are reduced by the increasing values of *R*_0_. The largest deviations observed are in the 60+ age class, with 28% against 16% of the average epidemic size obtained for *R*_0 _= 1.5 with GLEaM and with the agent-based model, respectively; 40% against 27% for *R*_0 _= 1.9; and 49% against 35% for *R*_0 _= 2.3. This is indeed the age class with the most marked difference in household structure and workplace habits that cannot be taken into consideration in the metapopulation level, thus generating the largest discrepancy between the two models.

## Discussion and conclusions

We studied a structured metapopulation model and an agent-based model to provide a side-by-side comparison of the modeling frameworks and assess the epidemic predictions that they can achieve. Starting from a shared parameterization of the disease progression and using identical initial conditions, we investigated and quantified similarities and differences in the results at different scales of resolution, and related those to the assumptions of the frameworks and to their integrated data. We found the two models to display a very good agreement in the timing of the epidemic, with a very limited variation in the time of the simulated epidemic activity peaks. In the metapopulation approach the fraction of the population affected by the epidemic is larger (by 5% to 10%) than in the agent-based approach. This difference is due to the assumption of homogeneity and thus the lack of detailed structure of contacts (besides the age structure) in the metapopulation approach with respect to the agent-based approach.

Our results highlight advantages and disadvantages of using the two approaches. On one side the detailed mobility networks considered in the metapopulation scheme provide an accurate description of the spreading pattern of the unfolding epidemic, identifying the major channels of transportation responsible for spreading the disease at the global level and quantifying the seeding events. On the other side, detailed estimations of the impact of the disease at a more local level are hampered by the lower level of detail contained in the metapopulation modeling scheme. The agent-based approach is extremely detailed but suffers from the difficulties in gathering high confidence datasets for most regions of the world. The good match between the two approaches in predicting the geotemporal spreading pattern of an epidemic demonstrates the feasibility of a hybrid approach that combines and integrates the two modeling schemes. Thanks to the heterogeneity of the transportation network, the spatio-temporal spread of an epidemic could be predicted at the global scale by employing a metapopulation approach. Taking advantage of the explicit representation of individuals in the model, the impact at a more local scale and the effects of individually-targeted interventions in specific areas could be predicted by employing an agent-based approach.

## Competing interests

AV is consulting and has a research agreement with Abbott for the modeling of H1N1. The other authors declare no competing interests.

## Authors' contributions

All authors have contributed to conceive, design and carry out the study and draft the manuscript.

## Pre-publication history

The pre-publication history for this paper can be accessed here:

http://www.biomedcentral.com/1471-2334/10/190/prepub

## Supplementary Material

Additional file 1 **Supplementary information comparing large-scale computational approaches to epidemic modeling: Agent-based versus structured metapopulation models**. A single pdf file 22 pages, the figures are embedded in the pdf. Click here for file
