# Supplementary material for: Comparing large-scale computational approaches to epidemic modeling: Agent-based versus structured metapopulation models
Source: BMC Infect Dis. 2010 Jun 29;10:190. doi: 10.1186/1471-2334-10-190 (PMC2914769; doi:10.1186/1471-2334-10-190)
Supplement: Additional file 1 — Supplementary information comparing large-scale computational approaches to epidemic modeling: Agent-based versus structured metapopulation models. A single pdf file 22 pages, the figures are embedded in the pdf. [file 1471-2334-10-190-S1.PDF]

Supplementary Information
Comparing large-scale computational approaches to epidemic modeling: agent-based versus structured metapopulation models

Marco Ajelli, Bruno Gonçalves, Duygu Balcan, Vittoria Colizza„ Hao Hu, José J. Ramasco, Stefano Merler & Alessandro Vespignani

I. AGENT-BASED MODEL

The agent-based model, adapted from that proposed by [1, 2], is a stochastic, spatially structured, individual based, discrete time epidemic simulator. The spread of the infection is modeled by explicitly considering transmission in households, schools and workplaces, and random contacts in the general population (see also [3, 5]). The model consists of (A) a sociodemographic model, in which individuals are co-located in households, schools, workplaces on the basis of census and commuting data, and (B) of an epidemiological model describing the spatio-temporal spread of the epidemic in the country.

A. Sociodemographic model

1. *Population data*

The population data of Italy ($56,995,744$ individuals) are obtained from the census [6] ($382,534$ census sections) and are hierarchically grouped by municipalities ($8,101$), provinces (103) and regions (20), according to the administrative borders of the study area (see Fig. 1 main paper). Number of individuals by municipality ranges from 33 to $2,546,804$ (Rome municipality) with an average of $7,035$ and a standard deviation of $39,326$. Only 42 municipalities contain more than $100,000$ individuals (6 of which contain more than $500,000$), while $1,971$ contain less than $1,000$ individuals (37 of which less than 100). The average surface of the administrative units is: $30\,km^2$ for municipalities, $129\,km^2$ for provinces and $661\,km^2$ for regions.

2. *Households*

The importance of considering realistic household groups in spatial studies of human diseases, such as influenza, is well known [7]. The explicit representation of household groups in the model allows testing the effectiveness of intervention options such as antiviral treatment of index cases and post-exposure prophylaxis of close contacts, which are considered key measures for containing/mitigating a new influenza pandemic [8]. Moreover, it is possible to evaluate the effectiveness of interventions at a spatial basis (e.g., the administration of antivirals to individuals living within a certain distance from a symptomatic case, quarantine measures), which can be crucial for containing an influenza pandemic at the source [1, 5]. Therefore, it arises the need of developing a realistic model of household groups. To such aim, we developed an heuristic model which matches marginal distributions of household size and population age structure, and maintains realistic generational age gaps within households (by avoiding randomly assigned ages to the households members), respecting as best as possible the actual mix of students, workers and inactive individuals.

Census data on age structure and household type and size [9] are used to randomly assign age and co-locate individuals in households. These data refer to the analysis of $19,227$ households, corresponding to approximately $0.1\,\%$ of the Italian households. Nine different types of household are considered: single, single with children, couple,

Table I: Age class of household heads in couples with children by household size (in percentage).

household size	age class						
	18–24	25–34	35–44	45–54	55–64	65–74	≥ 75
3	1.7	18.8	23.3	23.7	21.4	9.1	2
4	0.5	14.4	43.6	28.8	10.7	1.8	0.2
≥ 5	0.3	9.8	45.5	33.7	8.7	1.7	0.2

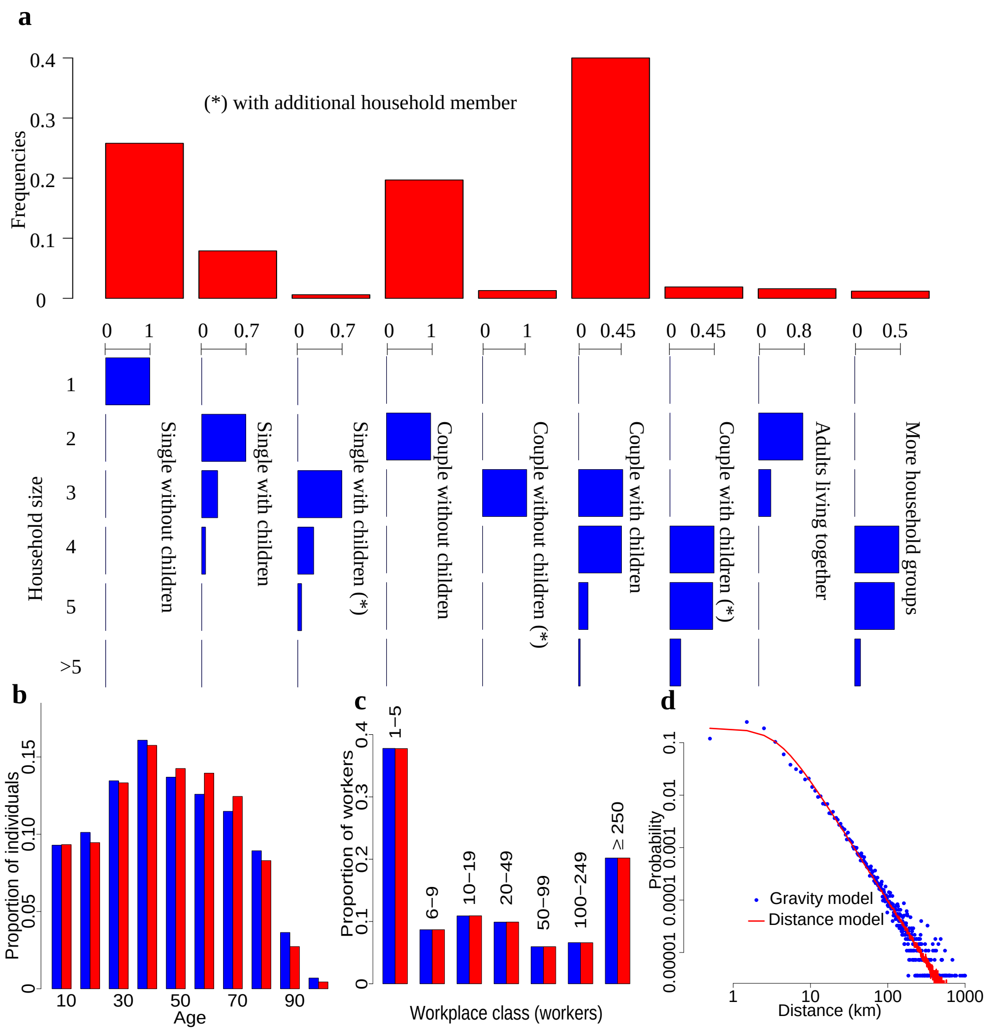

Figure 1: **a** Frequency distributions of household size for the different household types (in blue) and frequency distribution of the different household types (in red) considered in the model. The asterisk refers to an additional member aggregated to the family. **b** Age distribution from census data (blue) and simulated (red). **c** Proportion of workers for class of workplace from industry census data (blue) and simulated (red). **d** Probability density function of travel distances as obtained by using the gravity model (1) (in blue) compared with that obtained by using the the distance kernel (3) (in red).

couple with children, single with children and one additional member (e.g. elderly living with a son or a daughter), couple with children and one additional member, couple with one additional member, adults living together, more household groups. Frequency distribution of household sizes for the different household types are shown in Fig. 1a, together with the frequency distribution of the household types. The age of the household head for some of the different household types considered is reported in Table I (couples with children), Table II (couples without children) and Table III (singles without children). Note that the description "without children" applies either to families without children or with children which do not live with the parents anymore. The following additional constraints are also considered when assigning individuals to households:

C1 any household must contain at least 1 adult (age $\geq$ 18);

C2 the age of any child is between 18 and 43 years less than that of the youngest parent;

C3 spouses age differs by no more than 15 years.

A comparison between real and simulated age structure is reported in Fig. 1b. The algorithm used for generating household groups is shown in Fig. 2.

- For each municipality m
  - Let n_m be the number of individuals living in m
  - $n_a \leftarrow 0$
  - While $n_a < n_m$
    - determine household type $t \sim M(p_T)$, where $M(p_T)$ is a multinomial distribution with probabilities p_T given in Fig. 1a.
    - determine the household size $s \sim M_t(p_S(t))$, where $M(p_S(t))$ is a multinomial distribution with probabilities $p_S(t)$ given in Fig. 1a.
    - determine age class of household head $c \sim M(p_C(t,s))$, where $M(p_C(t,s))$ is a multinomial distribution with probabilities $p_C(t,s)$ given (for three household types) in Tab. I–III.
    - determine age of household head $a \sim M(p_A(c))$, where $M(p_A(c))$ is the multinomial distribution of the Italian age structure in the interval c.
    - determine age of other members (see additional constraints C1, C2 and C3).
    - $n_a \leftarrow n_a + s$

    EndWhile

  EndForeach

Figure 2: Pseudocode of the algorithm used for generating individuals, assigning age and co-locating them in households.

Table II: Age class of household head in couples without children.

age class	percentage
15–34	8
35–44	17.8
45–54	22.1
≥ 55	52.1

3. Employment

Demographic, school [10, 11] and industry [12] census data are used for randomly assigning an employment category to each individual on the basis of age. The Italian population in 2001 is structured as follows: $20,559,595$ workers, $11,360,556$ students and $25,084,274$ unemployed or retired. Students are deterministically assigned to a specific school type (6 types, from nursery school to university) on the basis of age. Attendance to school varies widely with age: it ranges from 14% in day care centers, to 90% in kindergartens, approximately 100% in primary and middle schools, 82% in high schools, 31% in university. Workers are assigned to a random workplace type (7 types, depending on the workplace size, i.e., the number of employees, see Fig. 1c). Students are grouped in classes, whose average size depends on the type. Specifically, the average size has been set to 20 in nursery schools, 40 in kindergartens, 19 in primary school, 21 in middle and high schools, 34 in universities [10, 11].

Table III: Age class of singles without children.

age class	percentage
15–24	1.5
25–44	24.2
45–64	21.8
≥ 65	52.5

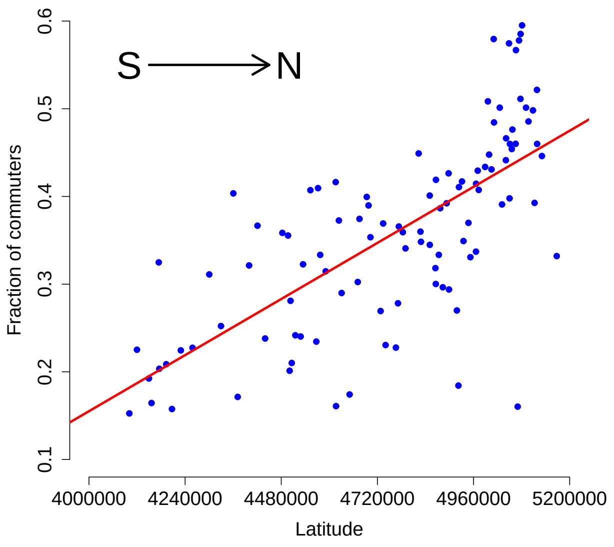

Figure 3: Proportion of commuters for the 103 Italian provinces as a function of the latitude of the provinces themselves.

4. Commuting

Commuting destination are assigned in order to fit available commuting data [6]. In particular, the proportion of individuals with age ≥ 15 working or attending school in the same municipality of residence is available for each municipality, together with the number of individuals traveling either to a municipality of the same province they live in, or outside the province but within the same region, or outside the region.

As a starting point, for determining the probability of commuting from municipality i to municipality j we employed a gravity model of the form [13]

$$C_{ij} = \theta \frac{N_i^{\tau_f} N_j^{\tau_t}}{d_{ij}^{\rho}} \; , \tag{1}$$

where N_i and N_j are the number of individuals living in municipality i and j respectively and d_{ij} is the distance between the two municipalities. θ is a proportionality constant, τ_f and τ_t tune the dependence of dispersal on donor and recipient sizes and ρ tunes the dependence on the distance.

The proportion of commuters (individuals traveling outside the municipality of residence for work or school) in Italy, however, varies significantly by province. In particular, the proportion of commuters drastically increases from South to North of Italy (see Fig. 3). The proportion of commuters varies from 15% to 60% and this variability does not depend on the size of the municipalities or on the distance among municipalities. Indeed, it depends on social factors and thus can not be explained by gravity law (1). We considered a model (1) with an additional constraint to obtain in each province the proportion of commuters as resulting from the available data.

The set of model parameters was optimized by searching for the set giving rise to a simulated population of commuters matching the available data on the number of individuals commuting within each province, each region or outside the region of residence. We obtained the following estimates: $\tau_f = 0.28$, $\tau_t = 0.66$ and $\rho = 2.95$ which are very close to those obtained in [13] for modeling travel destinations in the US at short distances (less than 119 km). Fig. 1d shows the resulting distance to work/school distribution, compared to actual data. Schools and workplaces are finally generated and located in municipalities to fit data on number and size and commuting data.

B. Epidemic model

1. *Transmission model*

The epidemic transmission is simulated by a stochastic, spatially structured, individual-based model. The epidemic is assumed to spread within the Italian population, accounting for ≈ 57 million individuals, kept constant during the simulations (i.e., without considering neither born/dead nor immigration/emigration processes). Individuals are explicitly represented in the model and are characterized by citizenship, household membership and school/workplace membership (if any). Households, schools and workplaces are located in an explicit geographic location.

Once the population is initialized, at any time t of the simulation (with time step $\Delta t = 1$ day), any susceptible individual i has a probability $p_i = 1 - e^{-\Delta t \cdot \lambda_i(t)}$ of becoming infected. The probability of becoming infected depends on the instantaneous risk of infection $\lambda_i(t)$, computed at any time step of the simulation. The risk of infection for each individual is defined as the sum of the risk factors coming from the different sources of infections considered, namely:

1. contacts with infectious members of the household (first term in Eq. (2)),
2. contacts with infectious individuals working in the same workplace or attending the same school (second term in Eq. (2)),
3. random contacts in the general population (third term in Eq. (2)).

$$\begin{aligned}\lambda_i \;=\; & \sum_{\{k=1,\dots,N \mid H_k = H_i\}} \frac{I_k c_k \beta_h}{n_{H_i}^{\alpha}} \\ + & \sum_{\{k=1,\dots,N \mid P_k^j = P_i^j\}} \frac{I_k c_k \beta_p^j \psi_p^j}{n_{P_i^j}} \\ + & \sum_{k=1}^{N} \frac{I_k c_k \beta_u f(d_{ik})}{\sum_{k=1}^{N} f(d_{ik})}\end{aligned} \tag{2}$$

The terms in Eq. (2) are defined as follows:

- N is the number of individuals of the population.
- H_i is the index of the household where individual i lives in.
- n_{H_i} is the number of individuals living in household H_i.
- P_i^j is the index of the place where individual i works/studies (if i is employed) and j identifies the place type (e.g., school, workplace).
- n_{P_i} is the number of individuals attending school/workplace P_i^j.
- $I_k = 1$ if individual k is infected, 0 otherwise.
- $c_k = 2$ for symptomatic cases (we assume the 67% of cases to be symptomatic), 1 otherwise. As discussed before, this choice is consistent with the one adopted in [1, 2, 14].
- α is a parameter determining the scaling of household transmission rates with household size. The value of $\alpha = 0.8$ is taken form [15], where it was introduced for fitting data on within households infections in past pandemics. Note that this value was previously used in many other influenza models (e.g., see [1, 2, 14]).
- $f(d_{ik})$ is the function in Eq. (3). It makes the transmission of the epidemic in the general community explicitly dependent on patterns of human mobility.
- β_h (expressed in day^{-1}) is the within–household transmission rate.

- β_p^j (in day^{-1}) is the within–school/workplace transmission rate, which depends on the type j of the place. Specifically, as in [2, 14], we assume that β_p^j is the same for all the school types and that it is two-times the transmission rate in the workplaces.
- β_u (in day^{-1}) is the transmission rate in the general community.
- ψ_p^j accounts for induced absenteeism and it is defined as follows: ψ_p^j is set to 0.1 for $j = 1, 2$, 0.2 for $j = 3, 4$, 0.25 for $j = 5$ and 0.5 for $j = 6, \ldots, 13$, 1 otherwise.

At any step t of the simulation, infected (exposed) individuals enter the infectious phase with probability $\Delta t \epsilon$, where ϵ^{-1} is the average latency period. When exposed individuals become infectious, they develop symptoms with probability 0.67. Finally, infectious individual recover from the infection with probability $\Delta t \mu$, where μ^{-1} is the average infectious period.

The third term of Eq. (2) accounts for the transmission due to random contacts with infectious individuals in the population. As in [1, 2], we assume that an infectious individual k can infect any susceptible individual i in the population and we assume that transmission depends explicitly on the distance between infectious individual k and susceptible individual i. The probability that an infectious individual k infects individual i is weighted by the kernel function

$$f(d_{ik}) = \frac{1}{1 + (d_{ik}/a)^b} \tag{3}$$

which is a decreasing function of the distance d_{ik} between the two individuals. Parameters a and b were optimized by employing Eq. (3) for generating a synthetic population of commuters such that the resulting distance to work/school distribution matches that obtained by employing the gravity model (1) (see Fig. 1d). The estimated parameters are $a = 3.8\,km$ and $b = 2.32$.

2. *Estimating* $\boldsymbol{R_0}$

For compartmental epidemic models, R_0 is defined as the dominant eigenvalue of the next-generation operator of the model [16]. For more structured models accounting for household and other kind of "small" groups (e.g., schools, classes, workplaces, etc.), the computation of R_0 is harder. Therefore, as in [2, 3, 14, 17], we compute R_0 by looking at the intrinsic growth rate of the simulated epidemics. Since we assume a fully susceptible population at the beginning of the simulations, the growth rate refers to the natural increase of the number of infected individuals during the initial phase of the epidemic, when the effects of susceptible depletion are negligible (and intervention strategies, if any, are not initiated).

Let us consider the following ordinary differential equations system, which is equivalent, from the epidemiological point of view, to the individual-based epidemic model:

$$\begin{cases} \dot{S} &= -\beta \frac{r_\beta I^a + I}{N} S \\ \dot{L} &= \beta \frac{r_\beta I^a + I}{N} S - \epsilon L \\ \dot{I} &= (1 - p_a)\epsilon L - \mu I \\ \dot{I}^a &= p_a \epsilon L - \mu I^a \\ \dot{R} &= \mu \left(I^a + I\right) \end{cases} \tag{4}$$

where the dependence of the variables on the time is omitted and

- S is the number of susceptible individuals;
- L is the number of latent individuals;
- I is the number of symptomatic infectious individuals;
- I^a is the number of asymptomatic infectious individuals;
- R is the number of recovered individuals;
- N is the total number of individuals in the population (which is assumed to be constant over time);
- β is the transmission rate of symptomatic individuals;

- r_β $(0 \le r_\beta \le 1)$ is the relative infectiousness of asymptomatic individual;
- p_a $(0 \le p_a \le 1)$ is the probability of becoming asymptomatic;
- ϵ^{-1} is the average latency period;
- μ^{-1} is the average infectious period;

The basic reproductive number of system (4), computed by employing the next–generation operator technique, is

$$R_0^{ode} \;=\; \frac{\beta}{\mu}\left[(1-p_a)+p_a r_\beta\right] \tag{5}$$

An equation depending explicitly on the initial exponential growth rate of the epidemic r can be obtained by linearizing the equations for latent and infectious individuals of system (4) at the disease-free equilibrium with $S = N$; in fact, r is essentially the dominant eigenvalue of this matrix [18]. The corresponding characteristic equation is the following:

$$(\mu+r)\left[(1-p_a)\epsilon\beta + p_a\epsilon r_\beta\beta - (\epsilon+r)(\mu+r)\right] \;=\; 0 \tag{6}$$

By solving Eq. (6) for the transmission rate β and by substituting the resulting value of β in Eq. (5) one obtains the following expression for R_0, depending only on r and known quantities:

$$R_0 \;=\; (1+r\mu^{-1})(1+r\epsilon^{-1}) \tag{7}$$

The intrinsic growth rate r was estimated by considering an epidemic spreading in Italy without considering imported cases. In order to (partially) eliminate the stochastic effects in the initial phase of the epidemic, we initialized the simulation with 100 infected individuals randomly chosen in the population. The intrinsic growth rate r and the resulting value of R_0 were obtained by fitting an exponential model $b_0 e^{rt}$ (for a certain constant b_0) to the cumulative number of symptomatic cases (during the initial phase of the simulated epidemic, lasting no less than 2 weeks). Finally, R_0 was computed by averaging over 100 experiments.

II. STRUCTURED METAPOPULATION MODEL

Here we present the detailed definition and data description of the global structured metapopulation model. The computational model is based on three data/model layers. The first layer is a data layer defining the census area and the subpopulation structure. The second one refers to human mobility model defined by the transportation and commuting networks characterizing the interactions and exchanges of individuals across subpopulations. The third layer is the epidemic dynamic model that defines the progression of the infectious disease inside each subpopulations.

A. Global Population and its Allocation

The population dataset was obtained from the Web sites of the "Gridded Population of the World" and the "Global Urban-Rural Mapping" projects [19, 20], which are run by the Socioeconomic Data and Application Center (SEDAC) of Columbia University. The surface of the world is divided into a grid of cells that can have different resolution levels. Each of these cells has assigned an estimated population value.

Out of the possible resolutions, we have opted for cells of 15×15 minutes of arc to constitute the basis of our model. This corresponds to an area of each cell approximately equivalent to a rectangle of $25 \times 25\,kms$ along the Equator. The dataset comprises $823,680$ cells, of which $250,206$ are populated. Since the coordinates of each cell center and those of the airports are known, the distance between the cells and the airports can be calculated. We have performed a Voronoi-like tessellation of the Earth surface assigning each cell to the closest airport that satisfies the following two conditions: (i) Each cell is assigned to the closest airport within the same country. And (ii), the distance between the airport and the cell cannot be longer than $200\,kms$. This cutoff naturally emerges from the distribution of distances between cells and closest airports, and it is introduced to avoid that in barely populated areas such as Siberia we can generate geographical census areas thousands of kilometer wide but with almost no population. It also corresponds to a reasonable upper cutoff for the ground traveling distance expected to be covered to reach an airport before traveling by plane.

Before proceeding with the tessellation, we need to take into account that some urban areas include more than one airport. For instance, London has up to six airport, Paris has two, and New York City has three. Our aim is to build a

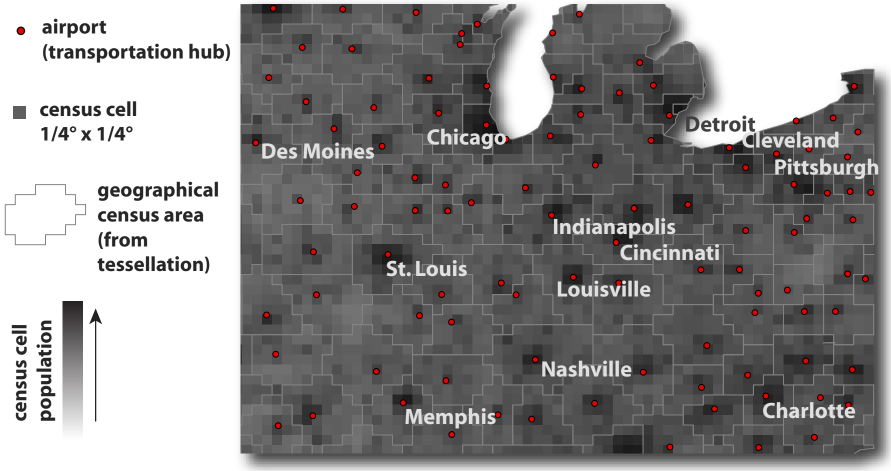

Figure 4: Population database and Voronoi tessellation around main transportation hubs. The world surface is represented in a grid-like partition where each cell - corresponding to a population estimate - is assigned to the closest airport. Geographical census areas emerge that constitute the sub-populations of the metapopulation model.

metapopulation model whose subpopulations correspond to the geographical census areas obtained from tessellation. Inside these geographical census areas a homogeneous mixing is assumed. The groups of airports that serve the same urban area need therefore to be aggregated since the mixing within the given urban area is expected to be high and cannot be represented in terms of separated subpopulations for each of the airports serving the same city. We have searched for groups of airports located close to each other and we manually processed the identified groups of airports to select those belonging to the same urban area. The airports of the same group are then aggregated in a single "super-hub". An example with the final result of the Voronoi tessellation procedure with cells and airports can be seen in Figure 4. The geographical census areas become thus the basic subpopulations of our metapopulation model. Their connections will determine the geographical spreading of an hypothetical epidemic. The air transportation is already integrated in the model, but a further step must be taken in order to also include ground transportation in a realistic way.

B. World Airport Network

The World Airport Network (WAN) is composed of 3,362 commercial airports indexed by the International Air Transport Association (IATA) that are located in 220 different countries. The database contains the number of available seats per year for each direct connection between two of these airports. The coverage of the dataset is estimated to be 99% of the global commercial traffic. The WAN can be seen as a weighted graph comprising 16,846 edges whose weight, $\omega_{j\ell}$, represents the passenger flow between airports j and ℓ. The network shows a high degree of heterogeneity both in the number of destinations per airport and in the number of passengers per connection [21–24].

C. Commuting Networks

Our commuting databases have been collected from the Offices of Statistics of 29 countries in 5 populated continents. The full dataset comprehends more than 80,000 administrative regions and over five million commuting flow connections between them (see Tables IV for details). The definition of administrative unit and the granularity level at which the commuting data are provided enormously vary from country to country. For example, most European countries adhere to a practice that ranks administrative divisions in terms of geocoding for statistical purposes, the so called Nomenclature of Territorial Units for Statistics (NUTS). Most countries in the European Union are partitioned into three NUTS levels which usually range from states to provinces. The commuting data at this level of resolution is therefore strongly coarse-grained. In order to have a higher geographical resolution of the commuting datasets that could match the resolution scale of our geographical census areas, we looked for smaller local administrative units (LAU) in Europe. The US or Canada report commuting at the level of counties. However, even within a single country

Table IV: Commuting networks in each continent. Number of countries (N_c), number of administrative units (V) and inter-links between them (E) are summarized.

Continent	N_c	V	E
Europe	17	65,880	4,490,650
North America	2	6,986	182,255
Latin America	5	4,301	102,117
Asia	3	2,732	323,815
Oceania	2	746	30,679
Total	29	80,645	5,129,516

the actual extension, shape, and population of the administrative divisions are usually a consequence of historical reasons and can be strongly heterogeneous.

Such heterogeneity renders the efforts to define a universal law describing commuting flows likely to fail. The mobility behavior might indeed result different across countries simply due to the country specific partition of the population into administrative boundaries. In order to overcome this problem, and in particular to define a data/driven short range commuting for GLEaM, we used the geographical census areas obtained from the Voronoi tessellation as the elementary units to define the centers of gravity for the process of commuting. This allows to deal with self-similar units across the world with respect to mobility as emerged from a tessellation around main hubs of mobility and not country specific administrative boundaries. We have therefore mapped the different levels of commuting data into the geographical census areas formed by the Voronoi-like tessellation procedure described above. The mapped commuting flows can be seen as a second transport network connecting subpopulations that are geographically close. This second network can be overlaid to the WAN in a multi-scale fashion to simulate realistic scenarios for disease spreading. The statistical properties of the commuting network at the level of the geographical census areas are reported in Ref. [25]. The network exhibits important variability in the number of commuters on each connection as well as in the total number of commuters per geographical census area. Being the census areas relatively homogeneous and self-similar allows us to estimate a gravity law that successfully reproduce the commuting data obtained across different continents, and provide us with estimations for the possible commuting levels in the countries for which such data is not available as in Ref. [25].

D. Epidemic dynamic model

Each geographical census area corresponds to a subpopulation in the metapopulation model, inside which we consider a Susceptible-Latent-Infectious-Recovered (SLIR) compartmental scheme, typical of influenza-like illnesses (ILIs), where each individual has a discrete disease state assigned at each moment in time. The contagion process, i.e. generation of new infections, is the only transition mechanism which is altered by short-range mobility, whereas all the other transitions between compartments are spontaneous and remain unaffected by the commuting. The rate at which a susceptible individual in subpopulation j acquires the infection, the so called force of infection λ_j, is determined by interactions with infectious persons either in the home subpopulation j or in its neighboring subpopulations on the commuting network.

Given the force of infection λ_j in subpopulation j, each person in the susceptible compartment (S_j) contracts the infection with $\lambda_j \Delta t$ and enters the latent compartment (L_j), where Δt is the time interval considered. Latent individuals exit the compartment with probability $\varepsilon\Delta t$, and transit to asymptomatic infectious compartment (I_j^a) with probability p_a or, with the complementary probability $1 - p_a$, become symptomatic infectious (see the compartmental flow diagram reported in the manuscript). Infectious persons with symptoms are further divided between those who can travel (I_j^t), probability p_t, and those who are travel-restricted (I_j^{nt}) with probability $1 - p_t$. All the infectious persons permanently recover with probability $\mu\Delta t$, entering the recovered compartment (R_j) in the next time step. All transitions and corresponding rates are summarized in Table V. In each subpopulation the variation of the number of individuals in each compartment $[m]$ can be written at any given time step as

$$X_j^{[m]}(t + \Delta t) - X_j^{[m]}(t) = \Delta X_j^{[m]} + \Omega_j([m]) \quad (8)$$

Table V: Transitions between compartments and their rates.

Transition	Type	Rate
$S_j \to L_j$	Contagion	λ_j
$L_j \to I_j^a$	Spontaneous	εp_a
$L_j \to I_j^t$		$\varepsilon(1-p_a)p_t$
$L_j \to I_j^{nt}$		$\varepsilon(1-p_a)(1-p_t)$
$I_j^a \to R_j$		μ
$I_j^t \to R_j$		μ
$I_j^{nt} \to R_j$		μ

where the term $\Delta X_j^{[m]}$ represents the change due to the compartment transitions induced by the disease dynamics and the transport operator $\Omega_j([m])$ represents the variations due to the traveling and mobility of individuals. The latter operator takes into account the long-range airline mobility and define the minimal time scale of integration to 1 day. The mobility due to the commuting flows is taken into account by defining effective force of infections by using a time scale separation approximations as detailed in the following sections.

E. Stochastic and discrete integration of the disease dynamics

In each subpopulation j, we define an operator acting on a compartment $[m]$ to account for all the transitions out of the compartment in the time interval Δt. Each element $\mathcal{D}_j([m],[n])$ of this operator is a random variable extracted from a multinomial distribution and determines the number of transitions from compartment $[m]$ to $[n]$ occurring in Δt. The change $\Delta X_j^{[m]}$ of a compartment $[m]$ in this time interval is given by a sum over all random variables $\{\mathcal{D}_j([m],[n])\}$ as follows

$$\Delta X_j^{[m]} = \sum_{[n]} \{-\mathcal{D}_j([m],[n]) + \mathcal{D}_j([n],[m])\} \quad . \tag{9}$$

As a concrete example let us consider the dynamics of the latent compartment. There are three possible transitions from the compartment: transitions to the asymptomatic infectious, the symptomatic traveling and the non-traveling infectious compartments. The elements of the operator acting on L_j are extracted from the multinomial distribution

$$Pr^{Multin}(L_j(t), p_{L_j\to I_j^a}, p_{L_j\to I_j^t}, p_{L_j\to I_j^{nt}}) \tag{10}$$

determined by the transition probabilities

$$\begin{aligned} p_{L_j\to I_j^a} &= \varepsilon p_a \Delta t \quad , \\ p_{L_j\to I_j^t} &= \varepsilon(1-p_a)p_t \Delta t \quad , \\ p_{L_j\to I_j^{nt}} &= \varepsilon(1-p_a)(1-p_t)\Delta t \quad , \end{aligned} \tag{11}$$

and by the number of individuals in the compartment $L_j(t)$ (its size). All these transitions cause a reduction in the size of the compartment. The increase in the compartment population is due to the transitions from susceptibles into latents. This is also a random number extracted from a binomial distribution

$$Pr^{Bin}(S_j(t), p_{S_j\to L_j}) \tag{12}$$

given by the chance of contagion

$$p_{S_j\to L_j} = \lambda_j \Delta t \quad , \tag{13}$$

with a number of attempts given by the number of susceptibles $S_j(t)$. After extracting these numbers from the appropriate multinomial distributions, we can calculate the change $\Delta L_j(t)$ as

$$\Delta L_j(t) = L_j(t+1) - L_j(t) = -\left[\mathcal{D}_j(L,I^a) + \mathcal{D}_j(L,I^t) + \mathcal{D}_j(L,I^{nt})\right] + \mathcal{D}_j(S,L) \; . \tag{14}$$

F. The integration of the transport operator

The transport operator is defined by the airline transportation data and sets the integration time scale to 1 day. The number of individuals in the compartment $[m]$ traveling from the subpopulation j to the subpopulation ℓ is an integer random variable, in that each of the $X_j^{[m]}$ potential travelers has a probability $p_{j\ell} = w_{j\ell}/N_j$ to go from j to ℓ. In each subpopulation j the numbers of individuals $\xi_{j\ell}$ traveling on each connection $j \to \ell$ at time t define a set of stochastic variables which follows the multinomial distribution

$$P(\{\xi_{j\ell}\}) = \frac{X_j^{[m]}!}{(X_j^{[m]} - \sum_\ell \xi_{j\ell})! \prod_\ell \xi_{j\ell}!}(1 - \sum_\ell p_{j\ell})^{(X_j^{[m]} - \sum_\ell \xi_{j\ell})} \prod_\ell p_{j\ell}^{\xi_{j\ell}}, \tag{15}$$

where $(1 - \sum_\ell p_{j\ell})$ is the probability of not traveling, and $(X_j^{[m]} - \sum_\ell \xi_{j\ell})$ identifies the number of non traveling individuals of the compartment $[m]$. We use standard numerical subroutines to generate random numbers of travelers following these distributions. The transport operator in each subpopulation j is therefore written as

$$\Omega_j([m]) = \sum_\ell (\xi_{\ell j}(X_\ell^{[m]}) - \xi_{j\ell}(X_j^{[m]})), \tag{16}$$

where the mean and variance of the stochastic variables are $\langle \xi_{j\ell}(X_j^{[m]}) \rangle = p_{j\ell} X_j^{[m]}$ and $\mathrm{Var}(\xi_{j\ell}(X_j^{[m]})) = p_{j\ell}(1 - p_{j\ell})X_j^{[m]}$. Direct flights as well as connecting flights up to two-legs flights can be considered. It is worth remarking that on average the airline network flows are balanced so that the subpopulation N_j are constant in time, e.g. $\sum_{[m]} \Omega_j([m]) = 0$.

G. Time-scale separation and the integration of the commuting flows

The Global Epidemic and Mobility (GLEaM) modeler combines the infection dynamics with long- and short-range human mobility. Each of these dynamical processes operates at a different time scale. For ILI there are two important intrinsic time scales, given by the latency period ε^{-1} and the duration of infectiousness μ^{-1}, both larger than 1 *day*. The long-range mobility given by the airline network has a time scale of the order of 1 *day*, while the commuting takes place in a time scale of approx. $\tau^{-1} \sim 1/3$ *day*. The explicit implementation of the commuting in the model thus requires a time interval shorter than the minimal time of airline transportation. To overcome this problem, we use a time-scale separation technique, in which the short-time dynamics is integrated into an effective force of infection in each subpopulation.

We start by considering the temporal dynamics of subpopulations linked only by commuting flows and evaluate the relaxation time to an equilibrium configuration. Consider the subpopulation j coupled by commuting to other n subpopulations. The commuting rate between the subpopulation j and each of its neighbors i will be given by σ_{ji}. The return rate of commuting individuals is set to be τ. Following the work of Sattenspiel and Dietz [26], we can divide the individuals original from the subpopulation j, N_j, between $N_{jj}(t)$ who are from j are located in j at time t and those, $N_{ji}(t)$, that are from j are located in a neighboring subpopulation i at time t. Note that by consistency

$$N_j = N_{jj}(t) + \sum_i N_{ji}(t). \tag{17}$$

The rate equations for the subpopulation size are then

$$\begin{aligned} \partial_t N_{jj} &= -\textstyle\sum_i \sigma_{ji} N_{jj}(t) + \tau \sum_i N_{ji}(t) \quad , \\ \partial_t N_{ji} &= \sigma_{ji} N_{jj}(t) - \tau N_{ji}(t) \quad . \end{aligned} \tag{18}$$

By using condition (17), we can derive the closed expression

$$\partial_t N_{jj} + (\tau + \sigma_j)\, N_{jj}(t) = N_j \tau \quad , \tag{19}$$

where σ_j denotes the total commuting rate of population j, $\sigma_j = \sum_i \sigma_{ji}$. $N_{jj}(t)$ can be expressed as

$$N_{jj}(t) = e^{-(\tau+\sigma_j)t} \left(C_{jj} + N_j \tau \int_0^t e^{(\tau+\sigma_j)s} ds \right) \quad , \tag{20}$$

where the constant C_{jj} is determined from the initial conditions, $N_{jj}(0)$. The solution for $N_{jj}(t)$ is then

$$N_{jj}(t) = \frac{N_j}{(1+\sigma_j/\tau)} + \left(N_{jj}(0) - \frac{N_j}{(1+\sigma_j/\tau)} \right) e^{-\tau(1+\sigma_j/\tau)t} \quad . \tag{21}$$

We can similarly solve the differential equation for the time dynamics of $N_{ji}(t)$

$$\begin{aligned} N_{ji}(t) \;&=\; \frac{N_j \sigma_{ji}/\tau}{(1+\sigma_j/\tau)} - \frac{\sigma_{ji}}{\sigma_j} \left(N_{jj}(0) - \frac{N_j}{(1+\sigma_j/\tau)} \right) e^{-\tau(1+\sigma_j/\tau)t} \\ &+\; \left[N_{ji}(0) - \frac{N_j \sigma_{ji}/\tau}{(1+\sigma_j/\tau)} + \frac{\sigma_{ji}}{\sigma_j} \left(N_{jj}(0) - \frac{N_j}{(1+\sigma_j/\tau)} \right) \right] e^{-\tau t} \quad . \end{aligned} \tag{22}$$

The relaxation to equilibrium of N_{jj} and N_{ji} is thus controlled by the characteristic time $[\tau\,(1+\sigma_j/\tau)]^{-1}$ in the exponentials. Such term is dominated by $1/\tau$ if the relation $\tau \gg \sigma_j$ holds. In our case, $\sigma_j = \sum_i \omega_{ji}/N_j$, that equals the daily total rate of commuting for the population j. Such rate is always smaller than one since only a fraction of the local population is commuting, and it is typically much smaller than $\tau \simeq 3 - 10\, day^{-1}$. Therefore the relaxation characteristic time can be safely approximated by $1/\tau$. This time is considerably smaller than the typical time for the air connections of one day and hence our approximation of considering the subpopulations $N_{jj}(t)$ and $N_{ji}(t)$ as relaxed to their equilibrium values,

$$N_{jj} = \frac{N_j}{1+\sigma_j/\tau} \quad \text{and} \quad N_{ji} = \frac{N_j \sigma_{ji}/\tau}{1+\sigma_j/\tau} \quad , \tag{23}$$

is reasonable. This approximation, originally introduced by Keeling and Rohani [27], allows us to consider each subpopulation j as having an effective number of individuals N_{ji} in contact with the individuals of the neighboring subpopulation i. In practice, this is similar to separate the commuting time scale from the other time scales in the problem (disease dynamics, traveling dynamics, etc.). While the approximation holds exactly only in the limit $\tau \to \infty$, it is good enough as long as τ is much larger than the typical transition rates of the disease dynamics. In the case of ILIs, the typical time scale separation between τ and the compartments transition rates is close to one order of magnitude or even larger. The Eqs. (23) can be then generalized in the time scale separation regime to all compartments $[m]$ obtaining the general expression

$$X_{jj}^{[m]} = \frac{X_j^{[m]}}{(1+\sigma_j/\tau)} \text{ and } X_{ji}^{[m]} = \frac{X_j^{[m]}}{(1+\sigma_j/\tau)} \sigma_{ji}/\tau \;, \tag{24}$$

where $\sigma_j = \sum_{i \in v(j)} \sigma_{ji}$ denotes the total commuting rate of j. Whereas $X_{jj}^{[m]} = X_j^{[m]}$ and $X_{ji}^{[m]} = 0$ for all the other compartments which are restricted from traveling. These expressions will be used to obtain the effective force of infection taking into account the interactions generated by the commuting flows.

H. Effective force of infection

The force of infection λ_j that a susceptible population of a subpopulation j sees can be decomposed into two terms: λ_{jj} and λ_{ji}. The component λ_{jj} refers to the part of the force of infection whose origin is local in j. While λ_{ji} indicates the force of infection acting on susceptibles of j during their commuting travels to a neighboring subpopulation i. The effective force of infection can be estimated by summing these two terms weighted by the probabilities of finding a susceptible from j in the different locations, S_{jj}/S_j and S_{ji}/S_j, respectively. Using the time-scale separation approximation that establishes the equilibrium populations in Eq. (24), we can write

$$\lambda_j = \frac{\lambda_{jj}}{1+\sigma_j/\tau} + \sum_i \frac{\lambda_{ji}\sigma_{ji}/\tau}{1+\sigma_j/\tau} \quad . \tag{25}$$

We will focus now on the calculation of each term of the previous expression. The force of infection occurring in a subpopulation j is due to the local infectious persons staying at j or to infectious individuals from a neighboring subpopulation i visiting j and so we can write

$$\lambda_{jj} = \frac{\beta_j}{N_j^*} \left[I_{jj}^{nt} + I_{jj}^{t} + r_\beta I_{jj}^{a} + \sum_i \left(I_{ij}^{nt} + I_{ij}^{t} + r_\beta I_{ij}^{a} \right) \right] \quad , \tag{26}$$

where β_j is introduced to account for the seasonality in the infection transmission rate (if the seasonality is not considered, it is a constant), and N_j^* stands for the total effective population in the subpopulation j. By definition, $I_{jj}^{nt} = I_j^{nt}$ and $I_{ji}^{nt} = 0$ for $j \neq i$. If we use the equilibrium values of the other infectious compartments (see Eq. (24)) we obtain

$$\lambda_{jj} \;=\; \frac{\beta_j}{N_j^*}\left[I_j^{nt} + \frac{I_j^t + r_\beta I_j^a}{1+\sigma_j/\tau} + \sum_i \frac{I_i^t + r_\beta I_i^a}{1+\sigma_i/\tau}\sigma_{ij}/\tau\right] \quad . \tag{27}$$

The derivation of λ_{ji} follows from a similar argument yielding:

$$\lambda_{ji} = \frac{\beta_i}{N_i^*}\left[I_{ii}^{nt} + I_{ii}^t + r_\beta I_{ii}^a + \sum_{\ell\in\upsilon(i)}\left(I_{\ell i}^{nt} + I_{\ell i}^t + r_\beta I_{\ell i}^a\right)\right] \quad , \tag{28}$$

where $\upsilon(i)$ represents the set of neighbors of i, and therefore the terms under the sum are due to the visits of infectious individuals from the subpopulations ℓ, neighbors of i, to i. By plugging the equilibrium values of the compartment into the above expression, we obtain

$$\lambda_{ji} \;=\; \frac{\beta_i}{N_i^*}\left[I_i^{nt} + \frac{I_i^t + r_\beta I_i^a}{1+\sigma_i/\tau} + \sum_{\ell\in\upsilon(i)} \frac{I_\ell^t + r_\beta I_\ell^a}{1+\sigma_\ell/\tau}\sigma_{\ell i}/\tau\right] \quad . \tag{29}$$

Finally, in order to have an explicit form of the force of infection we need to evaluate the effective population size N_j^* in each subpopulation j, i.e., the actual number of people actually staying at the location j. The effective population is $N_j^* = N_{jj} + \sum_i N_{ij}$, that in the time-scale separation approximation reads

$$N_j^* = I_j^{nt} + \frac{N_j - I_j^{nt}}{1+\sigma_j/\tau} + \sum_i \frac{N_i - I_i^{nt}}{1+\sigma_i/\tau}\sigma_{ij}/\tau \quad . \tag{30}$$

Note that in these equations all the terms with compartments have an implicit time dependence.
By inserting λ_{jj} and λ_{ji} into Eq. (25), it can be seen that the expression for the force of infection includes terms of zeroth, first and second order on the commuting ratios (i.e., σ_{ij}/τ). These three term types have a straightforward interpretation: The zeroth order terms represent the usual force of infection of the compartmental model with a single subpopulation. The first order terms account for the effective contribution generated by neighboring subpopulations with two different sources: Either susceptible individuals of subpopulation j having contacts with infectious individuals of neighboring subpopulations i, or infectious individuals of subpopulations i visiting subpopulation j. The second order terms correspond to an effective force of infection generated by the contacts of susceptible individuals of subpopulation j meeting infectious individuals of subpopulation ℓ (neighbors of i) when both are visiting subpopulation i. This last term is very small in comparison with the zeroth and first order terms, typically around two order of magnitudes smaller, and in general can be neglected.

I. Age Structure

The next step in perfecting our global model is to introduce a more detailed description of the populations within each census area. We start by distinguishing among different age groups with varying contact rates by using the results by Wallinga *et al* in [28]. In 2006, Wallinga *et al* [28] successfully measured the contact rates using a group of 1,813 Dutch survey participants. The contact matrix M, shown in Table VI, describes how many interactions an individual in one class has with individuals in a different age group. The columns correspond to the age of the survey participants, and the rows to the age of the people they interacted with. As an example, participants in the $20-39$ cohort reported having, on average, 5.70 conversations per week with adolescents in the $13-19$ group. Since we assume homogeneous mixing, the individuals within each group will interact freely among themselves. The contacts are not symmetric. This is easily explained if, for example, one considers children and adults. Children almost always live with adults, but adults do not always live with children. In order to obtain the effective rate of infection we must multiply the probability of infection by these rates since β is the rate at which a susceptible will become infected after contact with another infected person. However, we must still account for the reciprocity or, in other words, for the fact that the total number of interactions between two age groups must be the same. That is, we must have:

$$m_{ab}N_b \equiv m_{ba}N_a \tag{31}$$

		age of survey participants					
		$1-5$	$6-12$	$13-19$	$20-39$	$40-59$	$60+$
age of contacts	$0-5$	12.26	2.28	1.29	2.50	1.15	0.83
	$6-12$	2.72	23.77	2.80	3.02	1.78	1.00
	$13-19$	2.00	3.63	25.20	5.70	4.22	1.68
	$20-39$	11.46	11.58	16.87	25.14	16.43	8.34
	$40-59$	3.59	4.67	8.50	11.21	13.89	7.48
	$60+$	1.94	1.95	2.54	4.25	5.59	9.19

Table VI: Contact matrix M. From 28.

Age group	Number of participants	Dutch population ($\times 1000$)
0	0	184
$1-5$	125	876
$6-12$	154	$1,265$
$13-19$	152	$1,642$
$20-39$	681	$4,857$
$40-59$	360	$3,312$
$60+$	341	$2,477$
Total	$1,813$	$14,614$

Table VII: Wallinga's population structure.

The symmetrized matrix values are given by $C_{ab} = m_{ab} \cdot N/N_a$, where N_a is the number of individuals in age group a and N is the total number of individuals. The values of N_a for both the survey participants and the entire Dutch population are given in Table VII. The symmetric matrix C is shown in Table VIII.

Wallinga's work considers only 6 age groups, while our data for the demographics of each country provided by the US Census Bureau [29] can be more detailed. We distribute people uniformly within each 5 year compartment to divide the age groups so that they fit Wallinga's picture.

Modifying the way the different populations interact with each other changes the epidemic spreads, requiring modifications to the R_0 calculation. Here we use the technique described in [16, 30] and extend it to the general age structure case of interest. Let us first define $\vec{x} = (x_1, \ldots, x_n)$ to be a vector containing the number of individuals in each infected compartment. In this particular case, we have 4 such compartments, say, $L = x_1$, $I^t = x_2$, $I^{nt} = x_3$ and $I^a = x_4$. The matrix, F, defining the rate of creation of new infected cases is then:

$$F \equiv \begin{pmatrix} 0 & \beta & \beta & r_\beta \beta \\ 0 & 0 & 0 & 0 \\ 0 & 0 & 0 & 0 \\ 0 & 0 & 0 & 0 \end{pmatrix} \tag{32}$$

This is easily interpreted: Latent cases (first row) are created (from susceptible) with rate β ($r_\beta \beta$) through interaction with $I^{t,nt}$ (I^a). Since these are the only ways in which the disease can spread through a Susceptible population, all other entries in the matrix are null. After infection occurs, the disease progresses through several stages as described by the matrix $V = (v_{ij})$ where the value of element v_{ab} is the number of individuals leaving compartment a to compartment b, minus the number of individuals following the opposite path. For pandemic flu, this matrix is given by:

$$V \equiv \begin{pmatrix} \epsilon & 0 & 0 & 0 \\ -(1-p_a)\, p_t \epsilon & \mu & 0 & 0 \\ -(1-p_a)(1-p_t)\, \epsilon & 0 & \mu & 0 \\ -p_a \epsilon & 0 & 0 & \mu \end{pmatrix} \tag{33}$$

Using these two matrices we can calculate the next generation matrix,

$$\mathcal{N} \equiv F V^{-1} \tag{34}$$

that describes the complete epidemic process. The interpretation of this matrix is relatively simple: F is the rate at which new infections are created and V^{-1} is the average duration of each infected compartment. The basic

age of contacts	age of survey participants: 1 − 5	6 − 12	13 − 19	20 − 39	40 − 59	60+
0 − 5	169.14	31.47	17.76	34.50	15.83	11.47
6 − 12	31.47	274.51	32.31	34.86	20.61	11.50
13 − 19	17.76	32.31	224.25	50.75	37.52	14.96
20 − 39	34.50	34.86	50.75	75.66	49.45	25.08
40 − 59	15.83	20.61	37.52	49.45	61.26	32.99
60+	11.47	11.50	14.96	25.08	32.99	54.23

Table VIII: Symmetrized contact matrix. From 28.

reproductive rate, R_0 is finally given by the maximum eigenvalue of this matrix that in a model without age structure reads as

$$R_0 = \lambda_{max}\left(\mathcal{N}\right) \equiv \frac{\beta}{\mu}\left[r_\beta p_a + (1 - p_a)\right] \tag{35}$$

.

In a model with age structure we have to consider the proliferation of infected compartments. In the case of the Wallinga age grouping we have 6 times as many infected compartments. Fortunately, the fact that we don't consider aging implies that individuals never move between compartments corresponding to different age groups, thus greatly simplifying the analysis. We define the new vector $\vec{x}^\dagger$ to be a concatenation of 6 vectors $\vec{x}$ each corresponding to a different age cohort. Due to the mixing between the different age groups, a susceptible individual in one group can become latent by interacting with an infectious person from any other group. In matrix notation, and using the previous definitions, the new infection matrix $F^\dagger$ is given by

$$F^\dagger = M \times F \tag{36}$$

where $\times$ represents the Kroenecker product. This result can be easily verified after a few simple algebraic manipulations. After the initial infection, the disease progresses as before with each age group being isolated from the others. The progression matrix $V^\dagger$ is then

$$V^\dagger = \mathcal{I} \times V \tag{37}$$

where $\mathcal{I}$ is the 6×6 identity matrix. The next generation matrix can now be written as

$$\mathcal{N}^\dagger = M \times FV^{-1} \tag{38}$$

Therefore, the new basic reproductive number can be written as a function of the previous one

$$R_0^\dagger = R_0 \cdot \lambda_{max}\left(M\right) \tag{39}$$

This formulation is easily generalizable for any number of age groups with only a very small numerical effort. In order to set the specific value of R_0 used in our simulations, we invert this expression and calculate the appropriate value of $\beta(R_0)$.

The previous discussion has focused only on a single population. Since we are interested in adding age structure to our global simulation, we must take in to account the different demographics of each country or census areas and their change in time. The way to adjust the contact matrix for a different populational structure is straightforward.

Using the definitions above, we can write

$$\Delta I_a = \beta \sum_b \frac{m_{ab}}{N_a} S_a I_b \equiv \beta \sum_b c_{ab} S_a I_b \tag{40}$$

to describe the increase in the number of people in compartment I_a in a basic SI model. If we define the fraction of individuals in compartment I_a as $\rho_{I_a} \equiv I_a/N$ we can rewrite this expression as

$$\Delta\rho_{I_a} = \beta\rho_{S_a} \sum_b C_{ab} \rho_{I_b} \tag{41}$$

where C_{ab} is the symmetric matrix defined above. Since this expression depends only on the relative fraction of individuals in each compartment and not on the details of how many people are actually in each compartment, we can safely conclude that C_{ab} is the matrix that must be kept constant for every population. From this we can calculate

$$C_{ab} \equiv \frac{m^\dagger_{ab}}{N^\dagger_a} N^\dagger \equiv C^\dagger_{ab} \tag{42}$$

or, in other words

$$m^{\dagger}_{ab} \equiv C_{ab} \frac{N^{\dagger}_a}{N^{\dagger}} \tag{43}$$

as the matrix that we must use in Eq. (39) and that will differ from country to country. Substituting in Eq. (40) we obtain

$$\Delta I_a = \beta \sum_b C_{ab} S_a I_b \tag{44}$$

where N is the total population for the population being considered and C_{ab} is the same for every population.

It should be noted that during the derivation of these expressions, and for the sake of clarity, we considered only a *single population*. To obtain the expression for the full force of infection including the mobility dynamics Eq. **??** must still be modified according to the prescription of Sec. **??**. This can be easily done by simply replacing every term of the form $\beta_i I_i$ by:

$$\beta_i \sum_b C_{ab} I^b_i \tag{45}$$

III. COMPARISON OF RESULTS

In this section we report the results obtained from the sets of simulations with the values $R_0 = 1.5$ and $R_0 = 2.3$ not reported in the main text. Figures 5 and 6 show the average incidence profiles at the level of the GLEaM geographical census areas. The differences observed in the peak amplitude and in the synchronization of the profiles obtained from the two models tend to decrease for higher values of R_0. This is also observed from Figures 7 and 8 where these differences are quantified with respect to the latitude, population size, and airport traffic of each census area. The observed trends are in agreement with the results obtained for $R_0 = 1.9$ reported in the manuscript. The relative difference of epidemic size decreases for increasing values of population and traffic, and is stable across different values of the latitude. The average delay observed in the agent-based model with respect to GLEaM in the Southern census areas of Italy is also observed. As discussed before, the differences are reduced by larger values of the reproductive number.

Finally, Figures 9 and 10 show the geographical pattern of the epidemic spread of the two models at the level of the Italian municipalities, analogous to the ones reported in the main text for $R_0 = 1.9$. The reported snapshots show the good agreement of the simulations also at this resolution scale.

A. Single population model as a null hypotesis

We performed numerical simulations of a simple compartmental model with no age/social/spatial structure, and compared the results obtained by this simple model with the ones obtained by GLEaM and the agent-based model. All the parameter values of transmission dynamics used in the single population model have been set equal to those of GLEaM and agent-based model. The population size of the single population model has been set to Italian population. At each time step the total number of visitors arriving at international airports of Italy has been provided as initial conditions by GLEaM to align the initial conditions across different models. In order to keep the Italian population constant over time, the same number of people chosen at random has been dropped from the population.

By removing all possible sources of structure in the population, the observed epidemic shows a larger peak attack rate than the ones obtained assuming spatial and age structure − as in GLEaM − or assuming an individual-based approach as in the agent-based model. Moreover, the time pattern is not in good agreement with the one predicted by GLEaM and the agent-based model. Figure 11 compares the incidence profiles of the models in specific census areas, namely, Trieste and Rome. As expected, the simple compartmental model cannot reproduce the spatio-temporal pattern of the epidemics, mainly it is in great anticipation or delay as opposed to the other two models.

[1] Ferguson NM, Cummings DA, Cauchemez S, Fraser C, Riley S, Meeyai A, Iamsirithaworn S, Burke DS: **Strategies for containing an emerging influenza pandemic in Southeast Asia**. *Nature* 2005, 437:209–214.

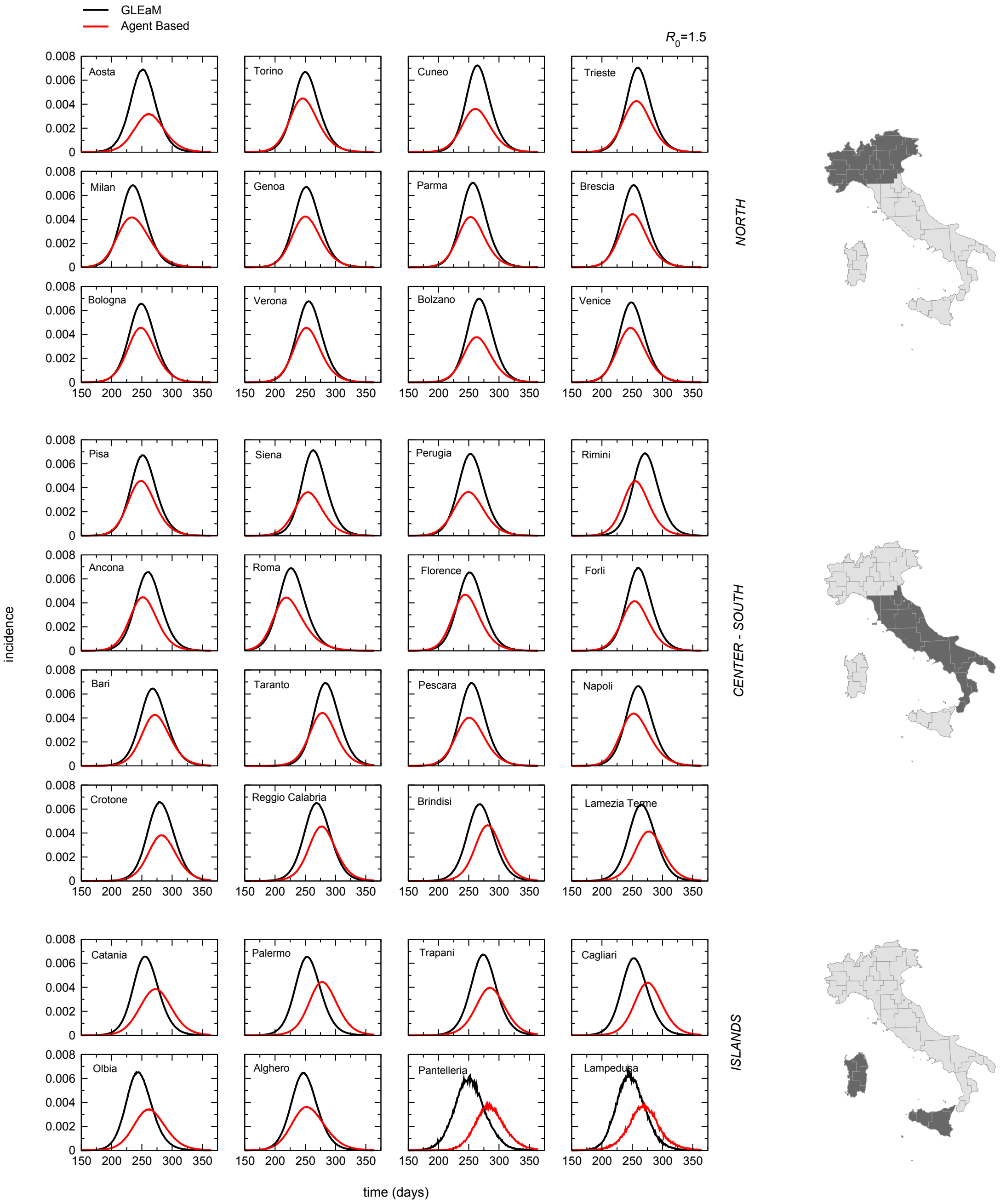

Figure 5: Average incidence profiles for GLEaM and the agent-based model at the level of the geographical census areas of GLEaM, for $R_0 = 1.5$. Time is expressed in days since the first importation of infectious individuals in Italy. Census areas are grouped into three regions – North (top), Center-South (center), and Islands (bottom).

[2] Ferguson NM, Cummings DA, Fraser C, Cajka JC, Cooley PC, Burke DS: **Strategies for mitigating an influenza pandemic**. *Nature* 2006, 442:448–452.

[3] Germann TC, Kadau K, Longini IMJ, Macken CA: **Mitigation strategies for pandemic influenza in the United States**. *Proc Natl Acad Sci USA* 2006, 103(15):5935–4.

[4] Glezen WP: **Emerging infections: pandemic influenza**. *Epidemiol Rev* 1996, 18(1):64–76.

[5] Longini IMJ, Nizam A, Xu S, Ungchusak K, Hanshaoworakul W, Cummings DA, Halloran ME: **Containing pandemic influenza at the source**. *Science* 2005, 309(5737):1083–1087.

[6] Italian Institute of Statistics (ISTAT) (2001b). XIV Censimento generale della popolazione e delle abitazioni. Availibale

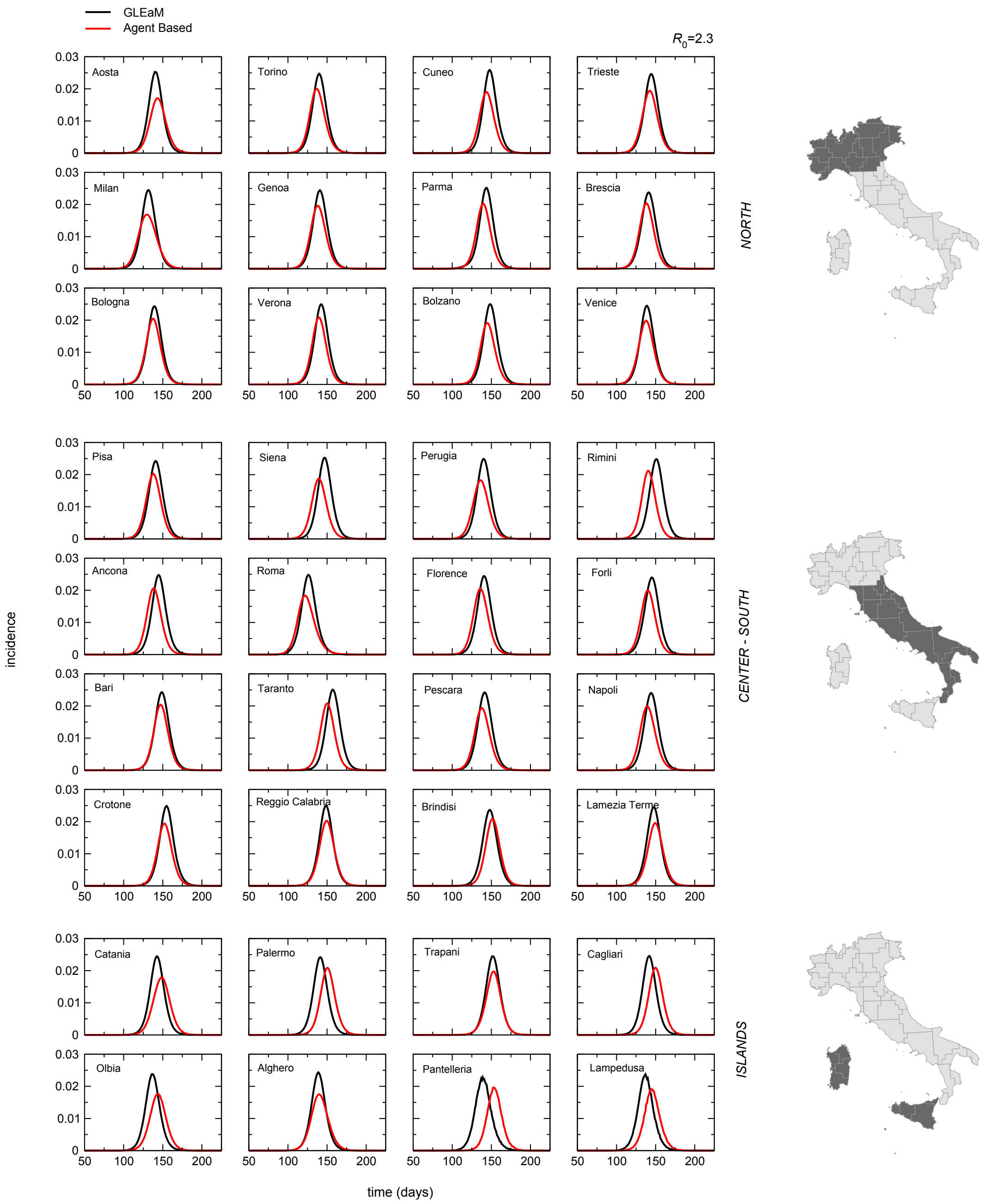

Figure 6: Average incidence profiles for GLEaM and the agent-based model at the level of the geographical census areas of GLEaM, for $R_0 = 2.3$. Time is expressed in days since the first importation of infectious individuals in Italy. Census areas are grouped into three regions – North (top), Center-South (center), and Islands (bottom).

on-line at `http://dawinci.istat.it/MD/`.

[7] Riley S: **Large-Scale Spatial-Transmission Models of Infectious Disease**. *Science* 2007, 316(5829):1298–1301.

[8] World Health Organization (2005). WHO global influenza preparedness plan. `http://www.who.int/csr/resources/publications/influenza/WHO_CDS_CSR_GIP_2005_5/en/`.

[9] Italian Institute of Statistics (ISTAT) (2003). Strutture familiari e opinioni su famiglia e figli. Availibale on-line at `http://www.istat.it/dati/catalogo/20060621_03/`.

[10] Italian Ministry of University and Reasearch (MIUR) (2005a). La scuola in cifre. Availibale on-line at `http://www.miur.it/ustat/documenti/pub2005/`.

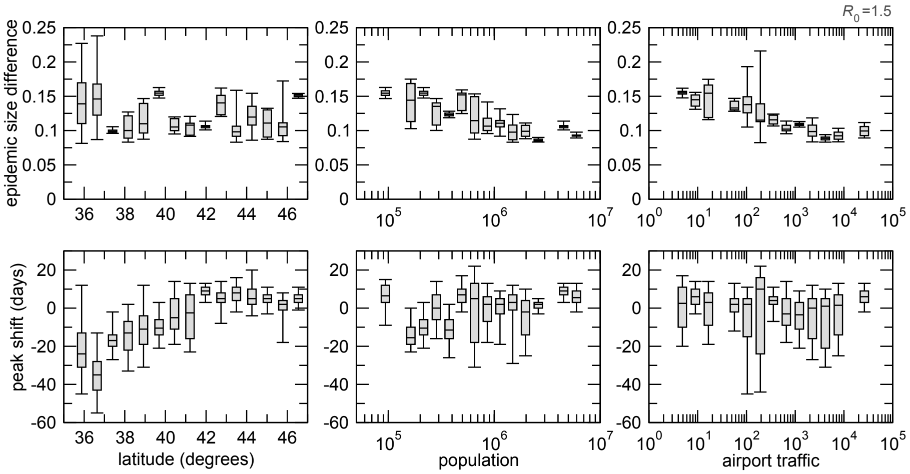

Figure 7: Difference of the epidemic size (top row) and peak shift (bottom row) between GLEaM and the agent-based model for $R_0 = 1.5$ at the level of geographical census areas as functions of: the latitude of the census area centroid (left); its population size (center); the traffic of its airport (right).

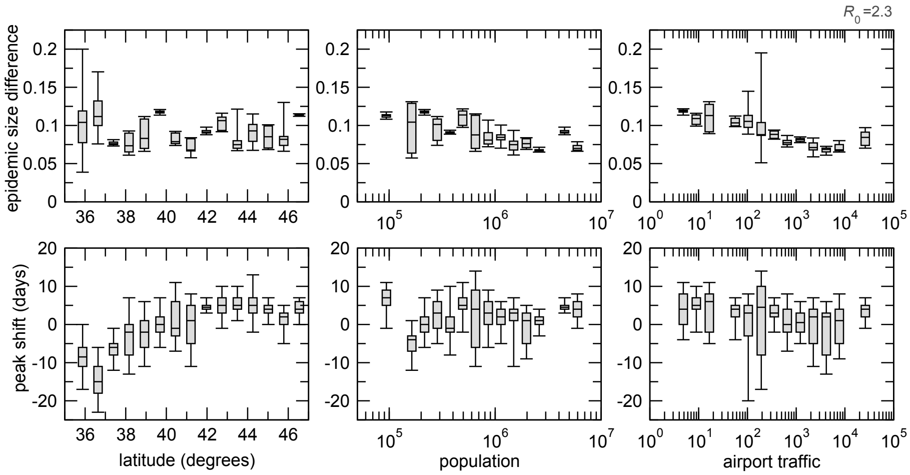

Figure 8: Difference of the epidemic size (top row) and peak shift (bottom row) between GLEaM and the agent-based model for $R_0 = 2.3$ at the level of geographical census areas as functions of: the latitude of the census area centroid (left); its population size (center); the traffic of its airport (right).

[11] Italian Ministry of University and Reasearch (MIUR) (2005b). L'università in cifre. Availibale on-line at `http://www.miur.it/ustat/documenti/pub2005/`.

[12] Italian Institute of Statistics (ISTAT) (2001a). VIII Censimento generale dell'industria e dei servizi . Availibale on-line at `http://dwcis.istat.it/cis/index.htm`.

[13] Viboud C, Bjørnstad O, Smith D, Simonsen L, Miller M, Grenfell B: **Synchrony, waves, and spatial hierarchies in the spread of influenza**. *Science* 2006, 312(5772):447–451.

[14] Ciofi degli Atti ML, Merler S, Rizzo C, Ajelli M, Massari M, Manfredi P, Furlanello C, Scalia Tomba G, Iannelli M: **Mitigation measures for pandemic influenza in Italy: an individual based model considering diffe rent scenarios**. *PLoS ONE* 2008, 3(3):e1790.

[15] Cauchemez S, Carrat F, Viboud C, Valleron A, Boelle P: **A Bayesian MCMC approach to study transmission of influenza: application to household longitudinal data**. *Stat Med* 2004, 23(22):3469–3487.

[16] Diekmann O, Heesterbeek J, Metz J: **On the definition and the computation of the basic reproduction ratio R0 in models for infectious diseases in heterogeneous populations**. *J Math Biol* 1990, 28(4):365–382.

[17] Ajelli M, Merler S: **The Impact of the Unstructured Contacts Component in Influenza Pandemic Modeling**. *PLoS ONE* 2008, 3(1):e1519.

[18] Chowell G, Hengartner N, Castillo-Chavez C, Fenimore P, Hyman J: **The basic reproductive number of Ebola and the effects of public health measures: the cases of Con go and Uganda**. *J Theor Biol* 2004, 229(1):119–126.

[19] Center for International Earth Science Information Network (CIESIN), Columbia University; and Centro Internacional de Agricultura Tropical (CIAT). The Gridded Population of the World Version 3 (GPWv3): Population Grids. Palisades, NY: Socioeconomic Data and Applications Center (SEDAC), Columbia University. `http://sedac.ciesin.columbia.edu/gpw`

[20] Center for International Earth Science Information Network (CIESIN), Columbia University; International Food Policy Research Institute (IFPRI); The World Bank; and Centro Internacional de Agricultura Tropical (CIAT). Global Rural-Urban Mapping Project (GRUMP), Alpha Version: Population Grids. Palisades, NY: Socioeconomic Data and Applications Center (SEDAC), Columbia University. `http://sedac.ciesin.columbia.edu/gpw`

[21] Barrat A, Pastor-Satorras R, Vespignani A: **The architecture of complex weighted networks**. *Proc. Natl. Acad. Sci. (USA)* 2004, 101:3747–3752.

[22] Colizza V, Barrat A, Barthelemy M, Vespignani A: **The role of the airline transportation network in the prediction and predictability of global epidemics**. *Proc. Natl. Acad. Sci. (USA)* 2006, 103:2015–2020.

[23] Colizza V, Barrat A, Barthelemy M, Vespignani A: **The modeling of global epidemics: Stochastic dynamics and predictability**. *Bull. Math. Biol.* 2006, 68: 1893–1921.

[24] Colizza V, Barrat A, Barthelemy M, Valleron A-J, Vespignani A: **Modeling the Worldwide Spread of Pandemic Influenza: Baseline Case and Containment Interventions**. *PLoS Med* 2007, 4(1):e13. doi:10.1371/journal.pmed.0040013.

[25] D. Balcan D, V. Colizza V, Gonçalves B, Hu H, Ramasco JJ, Vespignani A: **Multiscale mobility networks and the large scale spreading of infectious diseases**, *arXiv* 2009, 0907.3304.

[26] Sattenspiel L, Dietz K: (1995) **A structured epidemic model incorporating geographic mobility among regions**. *Mathematical Biosciences* 1995, 128:71–91.

[27] Keeling M J, Rohani P: (2002) **Estimating spatial coupling in epidemiological systems: a mechanistic approach**. *Ecology Letters* 2002, 5:20–29.

[28] Wallinga J, Teunis P, Kretzschmar M: **Using data on social contacts to estimate age-specific transmission parameters for respiratory-spread infectious agents**. *Am. J. Epi.* 2006, 164:936.

[29] International data base (idb). `http://www.census.gov/ipc/www/idb/`. Last accessed Jan 31, 2009.

[30] Heffernan JM, Smith RJ, Wahl LM: **Perspectives on the basic reproductive ratio**. *J. R. Soc. Interface* 2005, 2:281.

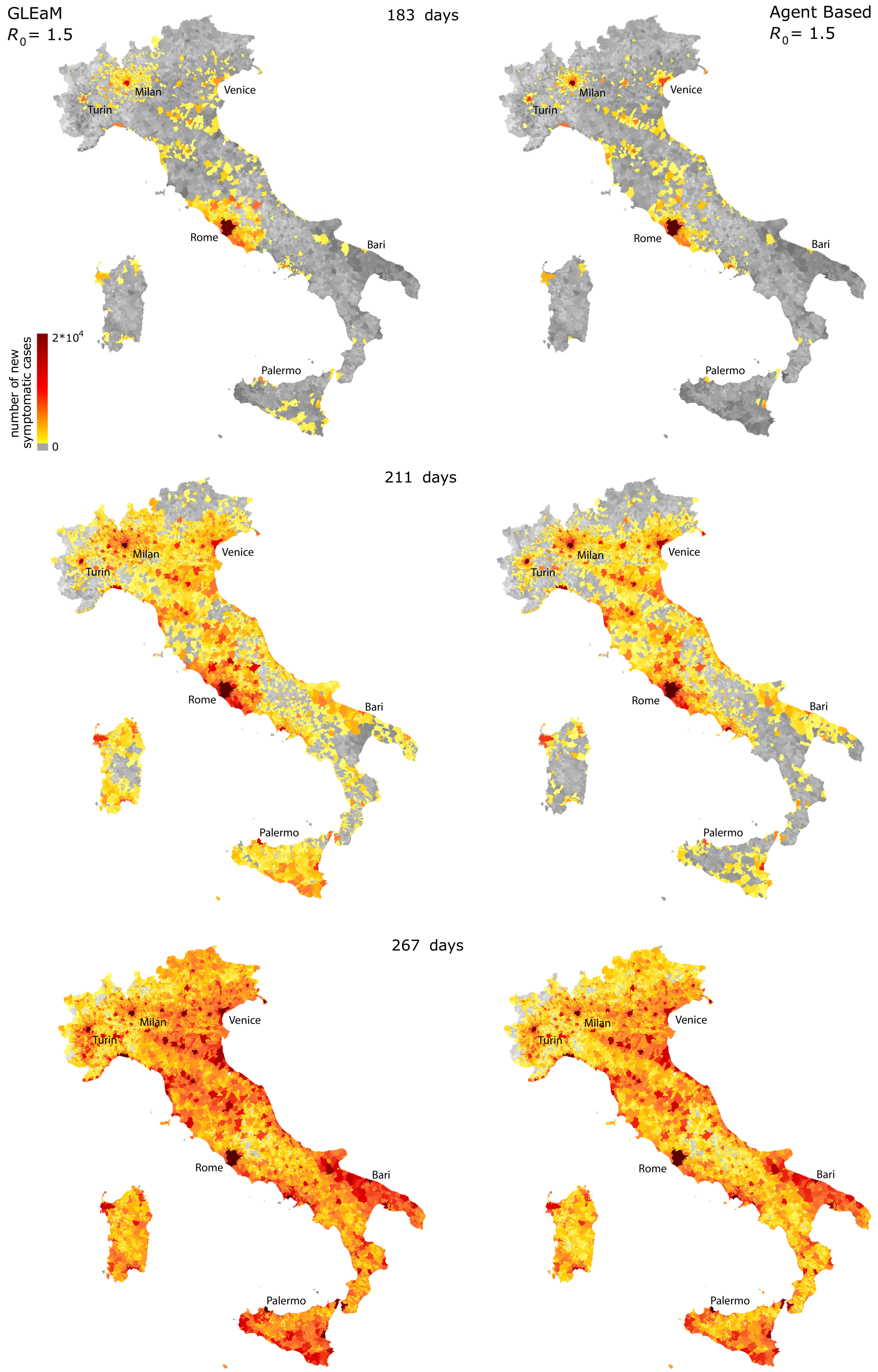

Figure 9: Comparison of the spatial epidemic spread in GLEaM (left) and the agent-based model (right) at three different snapshots of the simulation for $R_0 = 1.5$. Maps reproduce the average number of cases at the resolution scale of the Italian municipality.

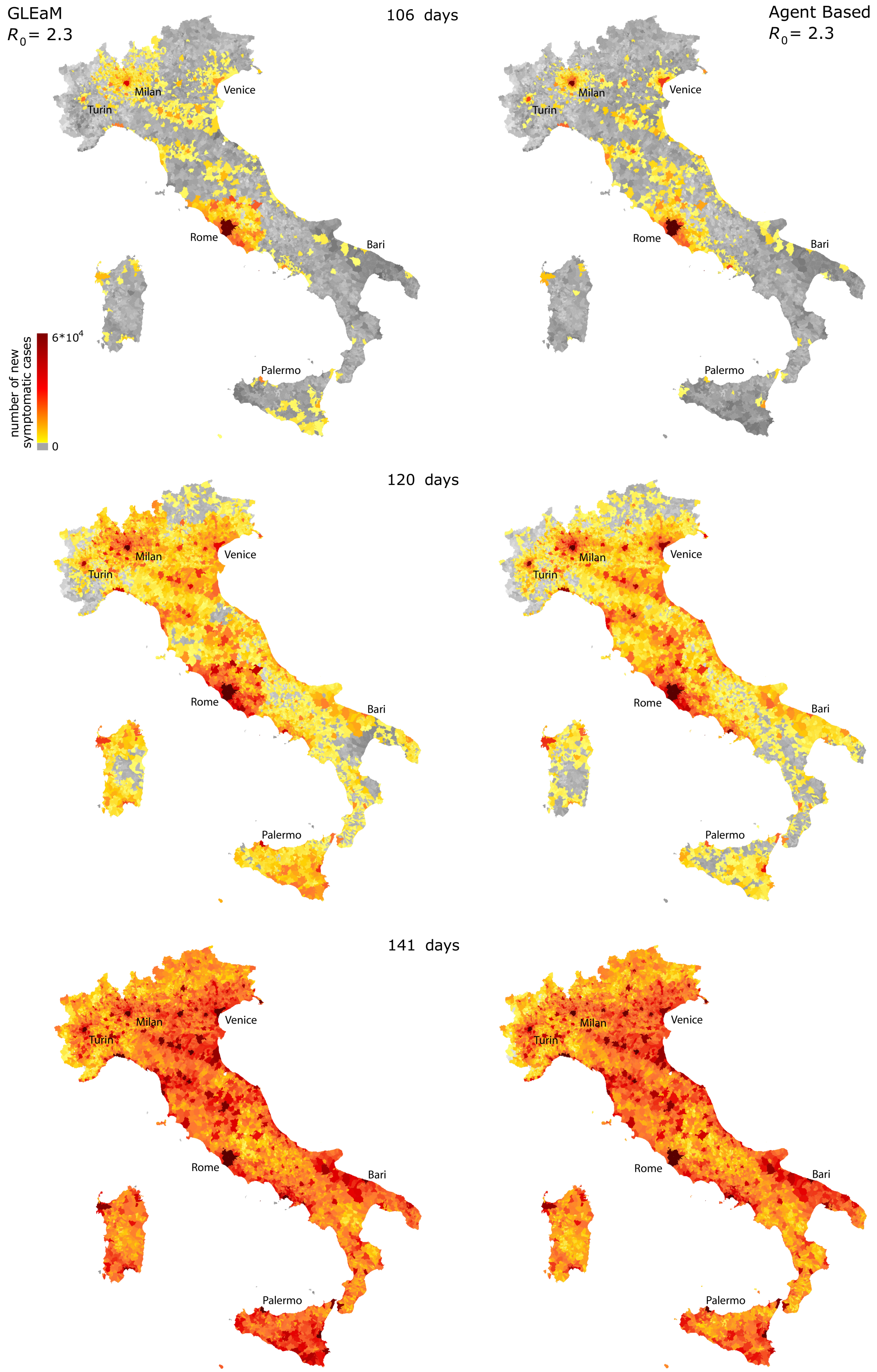

Figure 10: Comparison of the spatial epidemic spread in GLEaM (left) and the agent-based model (right) at three different snapshots of the simulation for $R_0 = 2.3$. Maps reproduce the average number of cases at the resolution scale of the Italian municipality.

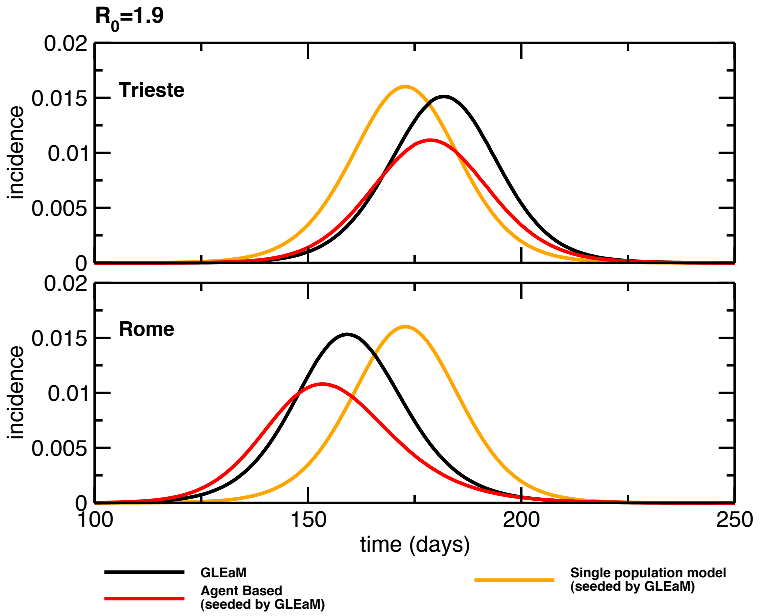

Figure 11: Comparison of the single population model with GLEaM and the agent-based model for $R_0 = 1.9$. As clearly seen, single population model, lack of spatial structure, is not able to recover the different timelines of the epidemics observed in different geographical areas.
